# Novel adiposity indices and their associations with all-cause and cardiovascular mortality in individuals with cardiovascular–kidney–metabolic syndrome stages 0–3: findings from a nationwide prospective cohort study

**DOI:** 10.3389/fendo.2025.1660210

**Published:** 2025-10-07

**Authors:** Hangyu Zhou, Yingying Xing, Tingting Wang, Tiantian Wang, Saiyaremu Xierzhati, Diliyaer Adili, Zhao Wang, Jie Li

**Affiliations:** The Cardiac and Panvascular Medicine Diagnosis and Treatment Center, People’s Hospital of Xinjiang Uygur Autonomous Region, Urumqi, Xinjiang, China

**Keywords:** cardiovascular–kidney–metabolic syndrome, obesity, novel adiposity, body mass index, all-cause mortality, cardiovascular mortality

## Abstract

**Background:**

Excess visceral fat drives CKM syndrome. This study assessed how newer obesity measures relate to death risk in early CKM (stages 0-3).

**Methods:**

This study included 26,899 participants with CKM stages 0–3 from the NHANES conducted between 2001 and 2018. Participants were grouped according to their baseline measurements of the adiposity indices (WWI, ABSI, WHtR, C-index, BRI, and BMI), which served as the exposure variables. Cox models, RCS curves, and two-stage Cox analyses were used to assess how novel obesity indices relate to mortality in this population. Subgroup analyses and sensitivity analyses evaluated risk differences across demographic groups and the robustness of the results. AUC, continuous NRI and IDI were used to compare the predictive performance of the novel indices and BMI for mortality.

**Results:**

The final analysis included a total of 26,899 participants. At the baseline, the gender distribution was 51.08% male, with an average age of 45.39 years. Compared with the lowest quartile group, the mortality rate was higher in the higher levels of the new obesity index groups.Novel indices significantly increased the risk of mortality. WWI showed the strongest link to all-cause death (HR:1.41, 95%CI:1.31 - 1.51) and cardiovascular death (HR 1.66,95%CI:1.39 - 1.99). ABSI, WWI, WHtR, and C-index had linear positive relationships with mortality. In contrast, BMI, BRI, and WHtR showed U-shaped associations with all-cause death (higher risk at both low and high values). The increased death risk linked to the new indices was greater in people aged 20–59 than in those ≥60. The incorporation of novel obesity biomarkers into the fully adjusted model significantly improved the predictive performance for adverse outcomes, as demonstrated by WWI, ABSI, and C-index. The continuous NRI values for these indices were 0.1831 (95% CI: 0.1289–0.1992), 0.2191 (0.1644–0.2877), and 0.1805 (0.1173–0.2398), with corresponding IDI values of 0.0356 (0.0193–0.0569), 0.0572 (0.0365–0.0839), and 0.0245 (0.0118–0.0432), respectively.

**Conclusion:**

Novel obesity index is closely associated with mortality risk in the early CKM population. Novel indices offer superior obesity assessment and mortality prediction in early CKM compared to BMI.

## Introduction

Cardiovascular diseases (CVDs), chronic kidney diseases (CKDs), and metabolic disorders (such as diabetes) constitute a significant burden on the global healthcare system. These conditions not only individually impose severe impacts on patient health but also present complex health challenges due to their shared risk factors and interrelated pathophysiological mechanisms (1–3). In 2023, the AHA formally defined cardiovascular–kidney–metabolic (CKM) syndrome as a multisystem disorder originating from dynamic pathophysiological cross-talk among metabolic abnormalities, CKD, and cardiovascular components. This systemic condition reflects the bidirectional biological mechanisms connecting these interrelated organ systems through shared molecular pathways and clinical manifestations (4). This definition underscores the multifaceted nature of CKM syndrome, highlighting the interconnectedness of these elements and their collective impact on overall health. CKM syndrome profoundly affects patient prognosis, resulting in poorer clinical outcomes and elevated mortality rates that exceed the cumulative risks associated with each individual disease component (5, 6). The AHA categorizes CKM syndrome into 5 stages (0-4) and emphasizes that stage 0–3 interventions should prioritize cardiovascular event prevention (4).

Previous studies have shown that obesity is a key factor in the progression of metabolic abnormalities and CVD (7, 8). Obesity exacerbates risk factors for cardiac and renal damage, such as diabetes and hypertension, thereby increasing the likelihood of cardiac and renal disease (9, 10). Excessive or dysfunctional adipose tissue is the most common cause of CKM pathogenesis. The proinflammatory and pro-oxidative products secreted by excessive adipose tissue further damage arterial, cardiac, and renal tissues (11, 12). The management of obesity is often closely associated with adverse health outcomes. For an extended period, BMI has been the primary metric employed to measure obesity. However, BMI has notable limitations in accurately assessing obesity indicators. It fails to differentiate between muscle and fat tissue and lacks precision in evaluating both the quantity and distribution of fat, including visceral and subcutaneous fat (13). Several studies have shown that BMI shows limited consistency as a prognostic biomarker for cardiovascular outcome, and there is a certain obesity paradox, where the risk of mortality is actually reduced in overweight or obese populations when BMI is used as an indicator for obesity assessment (14, 15). To address the limitations of conventional anthropometric measurements in characterizing regional adiposity patterns and total fat mass burden, novel adiposity indicators—including the waist–height ratio (WHtR), weight-adjusted waist circumference index (WWI), body shape index (ABSI), body roundness index (BRI), and conicity index (C-index)—have emerged as geometrically optimized metrics that demonstrate superior predictive validity for cardiometabolic risk stratification compared with traditional indices (16–18). Compared with BMI, these novel obesity markers, which comprehensively consider height, waist circumference (WC), weight and other associated metrics, can provide a more comprehensive understanding of the characteristics and distribution of visceral fat (19, 20). The latest research indicates that these novel obesity markers are positively correlated with all-cause mortality in patients with heart failure with preserved ejection fraction (HFpEF) (21). However, the prognostic associations between BMI and novel adiposity metrics with adverse outcomes remain insufficiently characterized across the early stages (stages 0-3) of CKM syndrome.

Therefore, we analyzed NHANES data to study how BMI and new obesity measures are related to death risk in early-stage CKM patients (stages 0-3) and to investigate the superiority of new obesity indices in obesity assessment and the prediction of adverse outcomes. This research will help guide weight management strategies for the CKM population to delay the occurrence of adverse outcomes.

## Methods

This prospective cohort study employed a nationally representative sample from the National Health and Nutrition Examination Survey (NHANES). Conducted biennially since 1999 by the Centers for Disease Control and Prevention (CDC)’s National Center for Health Statistics (NCHS), NHANES monitors the health and nutritional status of the U.S. civilian non-institutionalized population. Each year, approximately 5,000 randomly selected individuals participate through comprehensive health interviews (covering diet, socioeconomic factors, and health history), standardized physical/dental examinations at mobile centers, and laboratory tests performed by trained medical professionals. The NHANES uses a sophisticated multistage probability cluster sampling design with stratification weighting to achieve statistical generalizability to noninstitutionalized US civilian populations. This epidemiological surveillance system operates under stringent regulatory compliance, with secured Institutional Review Board (IRB) approval and the rigorous implementation of informed consent protocols that align with contemporary biomedical research ethics standards, including prospective documentation of voluntary participation through signed electronic consent forms. Before joining the research, all individuals provided a signed informed consent document. Detailed information about the design and data of the NHANES study is available on the official website (www.cdc.gov/nchs/nhanes/).

### Study population

**Figure 1:**
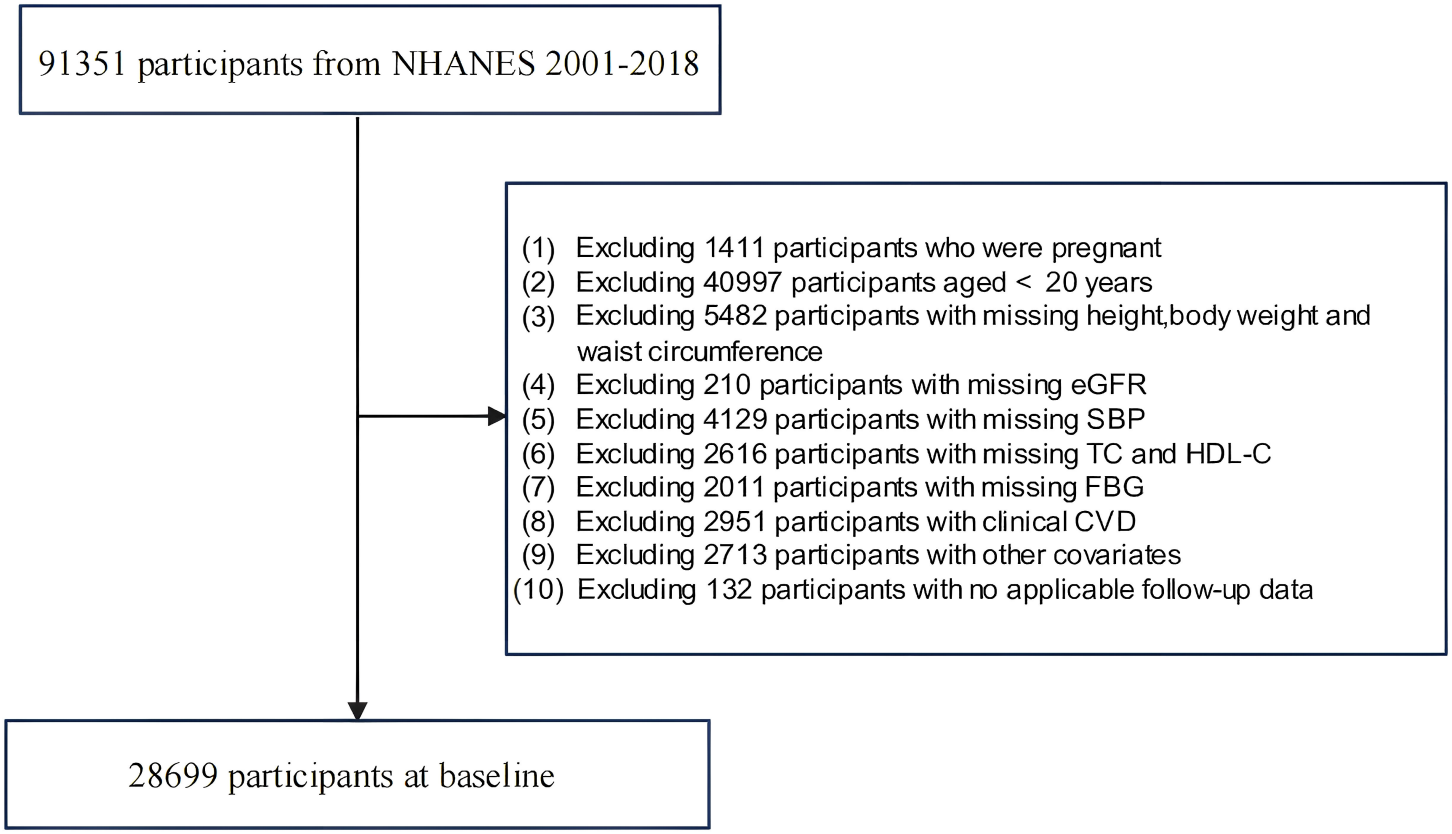
Flowchart of the study population.

Data from nine NHANES cycles spanning from 2001–2018 were employed in this study, which involved a total recruitment of 91,351 participants. Initially, participants who were pregnant or under 20 years of age were excluded. Those with missing data on BMI and novel obesity index-related variables were subsequently excluded. Additionally, owing to the requirements for CKM staging, participants lacking variables necessary for calculating the 10-year CVD risk via the Framingham equation were excluded. We then excluded participants with cardiovascular diseases. Finally, those with missing survival outcomes, inapplicable follow-up data, or incomplete baseline information were excluded. The final study sample included 28,699 adults aged 20 years and older, as detailed in **Figure 1**.

### Calculation of novel adiposity indices

The novel adiposity indices used in this study include the Body Roundness Index (BRI), A Body Shape Index (ABSI), Waist–Height Ratio (WHtR), Weight-adjusted Waist Circumference Index (WWI), and Conicity Index(C-index). These are comprehensive indicators that utilize data such as height, weight, and WC. The calculation formulas are as follows:


$$\text{BRI}=364.2-365.5\;\times \;{\left(1-\left[\text{WC}\left(\text{m}\right)/2\pi 2\right]/\left[0.5\times \text{height}\left(\text{m}\right)\right]2\right)}^{1/2};$$

$$\text{ABSI}=\text{WC}\left(\text{m}\right)/\left[\text{BM}{\text{I}}^{2/3}\left(\text{kg}/{\text{m}}^{2}\right)\;\times \text{ heigh}{\text{t}}^{1/2}\left(\text{m}\right)\right]\;\times \;1000;$$

WHtR=WC(cm)/height(cm);

$$\text{WWI}=WC\left(\text{cm}\right)/\text{weight}{\left(\text{kg}\right)}^{1/2};$$

$$\text{C}-\text{index}=\text{WC}\left(\text{cm}\right)/0.109{\left[\text{weight}\left(\text{kg}\right)/\text{height}\left(\text{m}\right)\right]}^{1/2};$$

### Definitions of CKM syndrome stages 0 to 3

As mentioned above, CKM syndrome emerges from interconnected dysfunctions involving cardiovascular impairment, CKD, and metabolic dysregulation. According to the CKM syndrome classification in the AHA Presidential Advisory Statement (4), participants were divided into distinct stages on the basis of their health status: Stage 0 signifies the nonexistence of CKM health risk factors; Stage 1 is marked by either excessive or dysfunctional obesity or the existence of prediabetes; Stage 2 entails the presence of metabolic risk factors or a moderate to high likelihood of CKD; and Stage 3 is recognized by the indication of subclinical CVD or being categorized in a very high-risk stage for CKD. Subclinical CVD is defined as having a significantly elevated predicted 10-year risk of CVD, as calculated via the standard Framingham formula (22). Renal function assessment was performed via the updated CKD-EPI algorithm (2021 creatinine-based) for eGFR determination and population stratification (23). The comprehensive staging criteria are provided in **Supplementary Table S1**.

### Definition of clinical outcome

Mortality data through 31 December 2019 were acquired from the NHANES open-access mortality linkage database, a resource constructed using death certificate documentation provided by the NCHS. The NHANES mortality data are linked to the National Death Index (NDI) using a hybrid deterministic-probabilistic methodology based on the Fellegi-Sunter paradigm. Deterministic matching first links records via exact Social Security Number (SSN) validation, while probabilistic scoring evaluates identifiers (e.g., name, birthdate, sex) for non-SSN records using agreement weights and Jaro-Winkler similarity. Matches are accepted if the probability exceeds 0.99, minimizing Type I/II errors (<2%). Follow-up time is calculated from interview/exam to death or December 31, 2019, with causes of death coded using ICD-9/ICD-10 standards. Public-use files perturb dates/causes to protect confidentiality, while restricted-access files retain full detail via NCHS Research Data Centers, as detailed in **Supplementary Materials**-Methods for linking mortality data. In this study, the median follow-up time was 107 months. A total of 132 participants were excluded due to missing survival outcomes or inapplicable follow-up data. All-cause mortality served as the principal study endpoint, with cardiovascular mortality constituting the secondary measure. Cardiovascular disease mortality refers specifically to deaths that occur as a result of heart disease. On the basis of the ICD-10 diagnostic coding protocols. The follow-up period commenced on the date of the interview and concluded on 31 December 2019 or at the time of death, whichever came first.

### Data collection

Data collection encompassed demographic characteristics, physical examinations, laboratory analyses, and lifestyle/medical histories, all assessed at baseline during the NHANES enrollment period (2001–2018). Demographic variables—including age, sex, race/ethnicity, marital status, and education level—were obtained via structured interviews. Physical examinations involved standardized measurements: height and weight (for BMI calculation) using a stadiometer and digital scale; waist circumference (WC) measured at the iliac crest; and systolic/diastolic blood pressure (SBP/DBP) recorded as the average of ≥3 readings after 5 minutes of rest. Laboratory testing, performed after a minimum 8-hour fast, included serum-based analyses: eGFR was calculated via the updated CKD-EPI algorithm (2021, creatinine-based); serum creatinine, uric acid, and BUN were measured enzymatically (creatinine: sarcosine oxidase; uric acid: uricase-PAP; BUN: urease/glutamate dehydrogenase); Fasting Blood Glucose (FBG) was quantified from sodium-fluoride plasma using the hexokinase method; Glycated Hemoglobin (HbA1c) was analyzed via high-performance liquid chromatography (HPLC); and lipid profiles (TG, TC, HDL-C, LDL-C) were assessed enzymatically—TG via glycerol phosphate oxidase-peroxidase (GPO-PAP), TC via cholesterol oxidase-peroxidase (CHOD-PAP), and HDL-C/LDL-C via homogeneous enzymatic methods (direct measurement). Individuals with a history of hypertension, along with those exhibiting an SBP of 130 mmHg or greater or a DBP of 80 mmHg or above at the start of the study, were categorized as having hypertension (24). People who have a documented history of diabetes, along with those exhibiting an FBG level of 7.0 mmol/L (126 mg/dL) or higher or an HbA1c level of 6.5% or more at the starting point, are classified as having diabetes (25). Additional lifestyle factors and medical histories were assessed through self-reported measures.

### Statistical analysis

In accordance with the NHANES guidelines, we accounted for the multistage sampling design by incorporating stratification variables, primary sampling units, and survey weights to maintain nationally representative estimates. Given the multicycle structure (n=9) implemented in this investigation, we calculate new weights. We categorized the BRI, WHtR, WWI, C-index, and ABSI into quartiles. Continuous variables conforming to a normal distribution are expressed as weighted means (± standard error). For nonnormally distributed continuous data, median values with interquartile ranges (interquartile ranges) are reported. Categorical variables are appropriately summarized via weighted proportions accompanied by 95% confidence intervals. Continuous variables that adhere to a normal distribution are examined with survey-weighted t tests, whereas those that do not meet this normality assumption are assessed via nonparametric rank-sum tests. Differences in the baseline characteristics of categorical variables were analyzed via survey-weighted chi-square tests. Survey-weighted univariate and multivariate Cox proportional hazards regression models were utilized, incorporating both the novel obesity index and BMI as continuous and categorical variables, to determine the HRs along with 95% CIs for overall mortality and cardiovascular-related deaths. Three regression models were developed. Taking into account the problem of collinearity, the variance inflation factor (VIF) for the covariates was assessed to guarantee that only those covariates with VIF values less than 5 were incorporated into the models, as detailed in **Supplementary Tables S2**-**S6** of the **Supplementary Material**. No violations of the proportional hazards assumption were detected via Schoenfeld residuals. The dose–response relationships between BRI, WHtR, WWI, C-index, ABSI, BMI and the endpoints (including all-cause mortality and cardiovascular mortality) were assessed via RCS curves with 4 knots. In cases of nonlinear relationships, a recursive method was utilized to detect possible inflection points, and threshold-specific associations were evaluated via segmented Cox regression models across identified breakpoints. Sensitivity analyses were conducted to verify the robustness of the results. Participants dying within the first 2 follow-up years were excluded to mitigate reverse causality. Re-ran all Cox regression models on the remaining cohort. Secondly, Fine-Gray proportional subdistribution hazard models were employed to assess the associations of the novel obesity index and BMI with cardiovascular mortality outcomes, with non-cardiovascular deaths treated as competing risk events. Stratified analyses examined effect modifications by demographic and clinical variables, including age, sex, lifestyle factors (alcohol consumption/smoking), cardiovascular and metabolic diseases, and CKM stages, with likelihood ratio tests used to evaluate subgroup interactions. ROC analyses with area under the curve (AUC) quantification were performed to assess the predictive performance of novel adiposity metrics versus conventional BMI for mortality outcomes. Continuous net reclassification improvement (NRI) and integrated discrimination improvement (IDI) were used to quantify the improvement in predictive performance after adding each new obesity index and BMI to the baseline model. If both indicators were positive and statistically significant (95% confidence interval did not include zero), it was considered an improvement in risk prediction ability.

Data were analyzed in R (v4.4.2) with two-sided tests; statistical significance was assigned at P < 0.05. More detailed statistical methods are provided in the **Supplementary Materials**.

## Results

### Demographic and clinical profiles of individuals across CKM syndrome stages 0–3

During the study period, 2,801 participants (9.76% of the cohort) died from all causes, with cardiovascular-related deaths accounting for 608 cases (2.12% of the total study population).

A comparative analysis of demographic and clinical characteristics across CKM stages 0–3 is summarized in **Table 1**. Most participants who faced outcomes related to all-cause mortality were older adults, male, non-Hispanic White individuals, well-educated individuals, and were primarily in stage 2 of CKM syndrome. The participants who experienced all-cause mortality presented elevated levels of uric acid, FBG, HbA1c, TG, LDL, TC, creatinine, BUN, BRI, ABSI, WWI, WHtR, C-index, WC, and SBP; increased rates of smoking, alcohol consumption, hypertension, and diabetes; and reduced DBP and eGFR.

**Table 1:** Characteristics of all-cause mortality in patients with CKM syndrome stages 0-3.

Variable	Total N=28699	Censored outcomes N=25898	All-cause death Outcomes N=2801	*P* value
Age, years	45.39 (0.20)	44.09 (0.20)	62.91 (0.42)	<0.001
Sex, n (%)				<0.001
Male	14979 (51.08)	13265 (50.65)	1714 (57.00)	
Female	13720 (48.92)	12633 (49.35)	1087 (43.00)	
Education,n (%)				<0.001
Less than high school	2570 (4.22)	2091 (3.77)	479 (10.33)	
High school	3902 (9.86)	3417 (9.51)	485 (14.64)	
Above high school	22227 (85.92)	20390 (86.73)	1837 (75.03)	
Marita, n (%)				<0.001
Married.	15201 (56.48)	13828 (56.88)	1373 (51.06)	
Unmarried.	13498 (43.52)	12070 (43.12)	1428 (48.94)	
Race,n (%)				<0.001
Non-Hispanic white	13405 (71.02)	11674 (70.36)	1731 (79.95)	
Non-Hispanic Black	5570 (9.56)	5017 (9.51)	553 (10.32)	
Mexican American	4892 (8.31)	4586 (8.66)	306 (3.61)	
Other Hispanic	2430 (5.16)	2298 (5.31)	132 (3.06)	
Other Race	2402 (5.95)	2323 (6.16)	79 (3.06)	
BMI, kg/m²	28.61 (0.08)	28.62 (0.08)	28.46 (0.17)	0.387
WC,cm	98.24 (0.19)	98.02 (0.20)	101.29 (0.39)	<0.001
SBP, mmHg	121.88 (0.17)	120.99 (0.17)	133.97 (0.50)	<0.001
DBP, mmHg	71.44 (0.15)	71.57 (0.16)	69.80 (0.33)	<0.001
Drinking,n (%)				<0.001
No	5686 (15.93)	4644 (14.68)	1042 (32.83)	
Yes	23013 (84.07)	21254 (85.32)	1759 (67.17)	
Smoke,n (%)				<0.001
No	23241 (81.24)	21092 (81.83)	2149 (73.29)	
Yes	5458 (18.76)	4806 (18.17)	652 (26.71)	
TC, mmol/L	5.09 (0.01)	5.08 (0.01)	5.20 (0.03)	<0.001
HDL-C, mmol/L	1.39 (0.00)	1.39 (0.01)	1.40 (0.01)	0.425
LDL-C, mmol/L	3.36 (0.01)	3.35 (0.01)	3.44 (0.03)	0.002
TG, mmol/L	1.70 (0.01)	1.69 (0.02)	1.81 (0.04)	0.002
HbA1c, n (%)	5.52 (0.01)	5.50 (0.01)	5.82 (0.02)	<0.001
FBG, mmol/L	5.37 (0.01)	5.33 (0.01)	5.93 (0.06)	<0.001
BUN, mmol/L	4.70 (0.02)	4.65 (0.02)	5.39 (0.06)	<0.001
Uric acid, ummol/L	320.68 (0.71)	319.41 (0.73)	337.88 (2.03)	<0.001
Serum creatinine,ummol/L	77.63 (0.19)	76.99 (0.19)	86.23 (0.81)	<0.001
eGFR, ml/min/1.73m2	95.20 (0.21)	95.74 (0.22)	87.88 (0.55)	<0.001
Hypertension, n (%)				<0.001
No	8586 (26.88)	7141 (25.26)	1445 (48.85)	
Yes	20113 (73.12)	18757 (74.74)	1356 (51.15)	
Diabetes,n (%)				<0.001
No	2716 (6.83)	2200 (6.20)	516 (15.44)	
Yes	25983 (93.17)	23698 (93.80)	2285 (84.56)	
CKM syndrome, n (%)				<0.001
Stage 0	1684 (6.92)	1631 (7.26)	53 (2.27)	
Stage 1	4935 (18.47)	4758 (19.19)	177 (8.79)	
Stage 2	20154 (69.22)	18026 (68.67)	2128 (76.69)	
Stage 3	1926 (5.39)	1483 (4.88)	443 (12.25)	
BRI,	5.16 (0.03)	5.13 (0.03)	5.66 (0.06)	<0.001
WHtR	0.58 (0.00)	0.58 (0.00)	0.60 (0.00)	<0.001
ABSI	81.07 (0.05)	80.83 (0.06)	84.30 (0.11)	<0.001
WWI	10.87 (0.01)	10.83 (0.01)	11.32 (0.02)	<0.001
C-index	1.30 (0.00)	1.29 (0.00)	1.35 (0.00)	<0.001

CKM syndrome, Cardiovascular-Kidney-Metabolic syndrome; BMI, body mass index; WC, waist circumference; eGFR, estimated glomerular filtration rate; SBP, systolic blood pressure; DBP, diastolic blood pressure; FBG, fasting blood glucose; HbA1c, glycated hemoglobin A1c; TG, triglycerides; TC, total cholesterol; HDL-C, high-density lipoprotein cholesterol; LDL-C, low-density lipoprotein cholesterol; BUN, blood urea nitrogen; BRI, body roundness index; ABSI,a body shape index; WHtR, waist-to-height ratio; WWI, weight-adjusted waist index; C-index, conicity index.

Continuous variables were presented as weighted means (standard error), and P values were obtained from t-test. Categorical variables were presented as frequency (percentages), and P values were obtained from the survey-weighted Chi-square test.

### Associations of BMI and novel adiposity indices with all-cause mortality and cardiovascular mortality

Survey-weighted multivariate regression and Cox proportional hazards modeling (three distinct models) were implemented to assess the associations of BMI and emerging adiposity indicators with mortality outcomes (all-cause/cardiovascular). When BMI and the novel obesity-related indices were treated as continuous variables, the findings indicated that, once potential confounding factors were accounted for, BMI did not exhibit a significant correlation with overall mortality or cardiovascular-related mortality in individuals suffering from CKM, as detailed in **Supplementary Material Table S7**. Other novel obesity indices are significantly associated with all-cause mortality and cardiovascular mortality, as detailed in **Tables 2**, **3**. The correlation between the WWI and all-cause mortality was found to be the most significant, with each WWI unit increase demonstrating 41% elevated mortality risk (HR = 1.41, 95% CI 1.31–1.51). The ABSI was associated with a 32% increase in mortality likelihood per 5-unit increment (HR = 1.32, 95% CI 1.24–1.39). C-index increments (per 0.1 units) correlated with 28% excess death probability (HR = 1.28, 95% CI 1.20–1.36). WHtR increases (0.1 units) corresponded to an 11% increased fatality risk (HR = 1.11, 95% CI 1.03–1.19). The BRI was associated with a 5% greater mortality risk per unit increase (HR = 1.05, 95% CI 1.02-1.08). The most significant association with cardiovascular mortality was found with WWI, where each unit increase led to a 66% increased risk of death (HR: 1.66, 95% CI 1.39–1.99). This was followed by the C-index, with each 0.1 unit increase associated with a 44% higher risk of death (HR: 1.444, 1.23–1.68). Each five-unit increase in ABSI was associated with a 38% greater risk of death (HR: 1.38,95% CI 1.22–1.56). The WHtR was associated with a 30% greater risk of death with each 0.1 unit increase (HR: 1.30, 95% CI 1.09–1.55). Finally, BRI was associated with an 11% greater risk of death with each unit increase (HR: 1.11,95% CI 1.05–1.20). We subsequently categorized BMIs into four groups, BMIs of 30 kg/m^2^, and divided the novel obesity indices into quartiles. Following the adjustment for possible confounding variables, the findings suggested that individuals with a BMI of 30 kg/m² presented a lower risk of all-cause mortality, as evidenced by HR (95% CI) values of 0.55 (0.40 - 0.73), 0.44 (0.32 - 0.60), and 0.46 (0.34 - 0.62), respectively. For more details, please refer to the **Supplementary Material**, **Supplementary Table S7**. Compared with the lowest quartile, the highest quartile of WWI, ABSI, and C-index were positively correlated with an increased risk of all-cause mortality, with HRs (95% CI) of 2.06 (1.71–2.48), 2.00 (1.71–2.34), and 1.65 (1.41–1.94), respectively. For details, please refer to **Table 2**. On the basis of BMI distribution, we defined four BMI categories: <18.5 kg/m² (underweight), 18.5–25 kg/m² (normal), 25–30 kg/m² (overweight), and ≥30 kg/m² (obese). Patients with a BMI of 25–30 kg/m^2^ were associated with a 0.49 (0.25–0.95) lower risk of cardiovascular mortality than those with a BMI <18.5 kg/m^2,^ as detailed in **Supplementary Table S7**. Relative to the lowest quartile, the upper quartiles of the WWI, ABSI, and C-index were associated with increased cardiovascular mortality risk: HR = 2.95 (1.77–4.93), 2.36 (1.49–3.72), and 2.35 (1.50–3.69), respectively, as detailed in **Table 3**. The BRI and WHtR were not significantly associated with all-cause or cardiovascular mortality, as detailed in **Tables 2**, **3**.

**Table 2:** Associations between the novel adiposity indices and all-cause mortality.

Variable	Model 1 HR (95% CI)	*P* value	Model 2 HR (95% CI)	*P* value	Model3 HR (95% CI)	*P* value
BRI						
Per 1 higher	1.14 (1.12 - 1.16)	<0.001	1.08 (1.05 - 1.10)	<0.001	1.05 (1.02 - 1.08)	<0.001
Quartile 1	1.00 (Reference)		1.00 (Reference)		1.00 (Reference)	
Quartile 2	1.31 (1.13 - 1.52)	<0.001	0.89 (0.76 - 1.04)	0.139	0.92 (0.78 - 1.08)	0.286
Quartile 3	1.72 (1.49 - 2.00)	<0.001	0.89 (0.76 - 1.04)	0.127	0.89 (0.75 - 1.05)	0.159
Quartile 4	2.48 (2.17 - 2.83)	<0.001	1.28 (1.10 - 1.49)	0.001	1.12 (0.95 - 1.33)	0.178
ABSI						
Per 5 higher	2.12 (1.93 - 2.33)	<0.001	1.44 (1.35 - 1.53)	<0.001	1.32 (1.24 - 1.39)	<0.001
Quartile 1	1.00 (Reference		1.00 (Reference		1.00 (Reference	
Quartile 2	1.53 (1.26 - 1.86)	<0.001	1.24 (1.02 - 1.51)	<0.001	1.19 (0.98 - 1.45)	0.077
Quartile 3	2.79 (2.30 - 3.39)	<0.001	1.72 (1.41 - 2.09)	<0.001	1.58 (1.30 - 1.92)	<0.001
Quartile 4	6.87 (5.90 - 8.00)	<0.001	2.47 (2.10 - 2.90)	<0.001	2.00 (1.71 - 2.34)	<0.001
WWI						
Per 1 higher	2.33 (2.19 - 2.47)	<0.001	1.55 (1.44 - 1.66)	<0.001	1.41 (1.31 - 1.51)	<0.001
Quartile 1	1.00 (Reference)		1.00 (Reference)		1.00 (Reference)	
Quartile 2	1.84 (1.54 - 2.19)	<0.001	1.29 (1.08 - 1.54)	0.004	1.30 (1.09 - 1.55)	0.004
Quartile 3	2.91 (2.51 - 3.37)	<0.001	1.51 (1.28 - 1.79)	<0.001	1.49 (1.26 - 1.77)	<0.001
Quartile 4	6.42 (5.52 - 7.47)	<0.001	2.37 (2.01 - 2.81)	<0.001	2.06 (1.71 - 2.48)	<0.001
WHtR						
Per 0.1 higher	1.39 (1.33 - 1.46)	<0.001	1.17 (1.10 - 1.25)	<0.001	1.11 (1.03 - 1.19)	<0.001
Quartile 1	1.00 (Reference)		1.00 (Reference)		1.00 (Reference)	
Quartile 2	1.31 (1.13 - 1.52)	<0.001	0.89 (0.76 - 1.04)	0.139	0.92 (0.78 - 1.08)	0.286
Quartile 3	1.72 (1.49 - 2.00)	<0.001	0.89 (0.76 - 1.04)	0.127	0.89 (0.75 - 1.05)	0.159
Quartile 4	2.48 (2.17 - 2.83)	<0.001	1.28 (1.10 - 1.49)	0.001	1.12 (0.95 - 1.33)	0.178
C-index						
Per 0.1 higher	2.06 (1.93 - 2.20)	<0.001	1.39 (1.30 - 1.48)	<0.001	1.28 (1.20 - 1.36)	<0.001
Quartile 1	1.00 (Reference)		1.00 (Reference)		1.00 (Reference)	
Quartile 2	1.63 (1.34 - 1.97)	<0.001	1.20 (1.00 - 1.45)	0.053	1.19 (0.98 - 1.44)	0.072
Quartile 3	2.70 (2.29 - 3.19)	<0.001	1.43 (1.20 - 1.71)	<0.001	1.39 (1.17 - 1.66)	<0.001
Quartile 4	5.38 (4.68 - 6.19)	<0.001	1.94 (1.65 - 2.27)	<0.001	1.65 (1.41 - 1.94)	<0.001

Model 1: No adjustments.

Model 2: Adjusted for age, sex, race.

Model 3: Adjusted for age, sex, race, marital status, education, uric acid, serum creatinine, BUN, HDL-C, LDL-C, TG, FBG, HbA1c, smoking, drinking, hypertension, diabetes.

HR, hazards ratio; CI, confidence interval. Other abbreviations, see **Table 1**.

**Table 3:** Associations between the novel adiposity indices and cardiovascular mortality.

Variable	Model 1 HR (95% CI)	*P* value	Model 2 HR (95% CI)	*P* value	Model3 HR (95% CI)	*P* value
BRI						
Per 1 higher	2.76 (2.45 - 3.11)	<0.001	1.16 (1.10 - 1.23)	<0.001	1.12 (1.05 - 1.20)	<0.001
Quartile 1	1.00 (Reference)		1.00 (Reference)		1.00 (Reference)	
Quartile 2	1.34 (0.97 - 1.84)	0.075	0.84 (0.61 - 1.16)	0.284	0.85 (0.61 - 1.18)	0.331
Quartile 3	1.99 (1.44 - 2.76)	<0.001	0.91 (0.65 - 1.29)	0.598	0.87 (0.60 - 1.26)	0.452
Quartile 4	3.36 (2.43 - 4.65)	<0.001	1.58 (1.13 - 2.23)	0.008	1.30 (0.86 - 1.96)	0.211
ABSI						
Per 5 higher	2.29 (2.02 - 2.60)	<0.001	1.51 (1.34 - 1.71)	<0.001	1.38 (1.22 - 1.56)	<0.001
Quartile 1	1.00 (Reference		1.00 (Reference		1.00 (Reference	
Quartile 2	1.85 (1.22 - 2.83)	0.004	1.41 (0.91 - 2.20)	0.124	1.35 (0.88 - 2.07)	0.165
Quartile 3	3.08 (1.95 - 4.85)	<0.001	1.68 (1.01 - 2.79)	0.044	1.53 (0.92 - 2.53)	0.100
Quartile 4	9.85 (6.73 - 14.41)	<0.001	2.91 (1.83 - 4.65)	<0.001	2.36 (1.49 - 3.72)	<0.001
WWI						
Per 1 higher	2.76 (2.45 - 3.11)	<0.001	1.85 (1.57 - 2.19)	<0.001	1.66 (1.39 - 1.99)	<0.001
Quartile 1	1.00 (Reference)		1.00 (Reference)		1.00 (Reference)	
Quartile 2	2.06 (1.41 - 3.02)	<0.001	1.38 (0.92 - 2.07)	0.119	1.39 (0.91 - 2.11)	0.130
Quartile 3	3.54 (2.41 - 5.20)	<0.001	1.70 (1.10 - 2.62)	0.016	1.70 (1.08 - 2.66)	0.021
Quartile 4	10.14 (6.99 - 14.69)	<0.001	3.43 (2.16 - 5.45)	<0.001	2.95 (1.77 - 4.93)	<0.001
WHtR						
Per 0.1 higher	1.61 (1.46 - 1.78)	<0.001	1.41 (1.22 - 1.63)	<0.001	1.30 (1.09 - 1.55)	<0.001
Quartile 1	1.00 (Reference)		1.00 (Reference)		1.00 (Reference)	
Quartile 2	1.34 (0.97 - 1.84)	0.075	0.84 (0.61 - 1.16)	0.284	0.85 (0.61 - 1.18)	0.331
Quartile 3	1.99 (1.44 - 2.76)	<0.001	0.91 (0.65 - 1.29)	0.598	0.87 (0.60 - 1.26)	0.452
Quartile 4	3.36 (2.43 - 4.65)	<0.001	1.58 (1.13 - 2.23)	0.008	1.30 (0.86 - 1.96)	0.211
C-index						
Per 0.1 higher	2.41 (2.12 - 2.75)	<0.001	1.59 (1.37 - 1.83)	<0.001	1.44 (1.23 - 1.68)	<0.001
Quartile 1	1.00 (Reference)		1.00 (Reference)		1.00 (Reference)	
Quartile 2	2.09 (1.45 - 3.01)	<0.001	1.46 (0.98 - 2.16)	0.062	1.43 (0.96 - 2.12)	0.079
Quartile 3	3.31 (2.23 - 4.93)	<0.001	1.56 (1.00 - 2.45)	0.051	1.51 (0.96 - 2.37)	0.071
Quartile 4	9.30 (6.42 - 13.47)	<0.001	2.85 (1.85 - 4.41)	<0.001	2.35 (1.50 - 3.69)	<0.001

Model 1: No adjustments.

Model 2: Adjusted for age, sex, race.

Model 3: Adjusted for age, sex, race, marital status, education, uric acid, serum creatinine, BUN, HDL-C, LDL-C, TG, FBG, HbA1c, smoking, drinking, hypertension, diabetes.

HR, hazards ratio; CI, confidence interval. Other abbreviations, see **Table 1**.

### Dose–response relationships of BMI and novel obesity markers with all-cause and cardiovascular mortality

**Figure 2:**
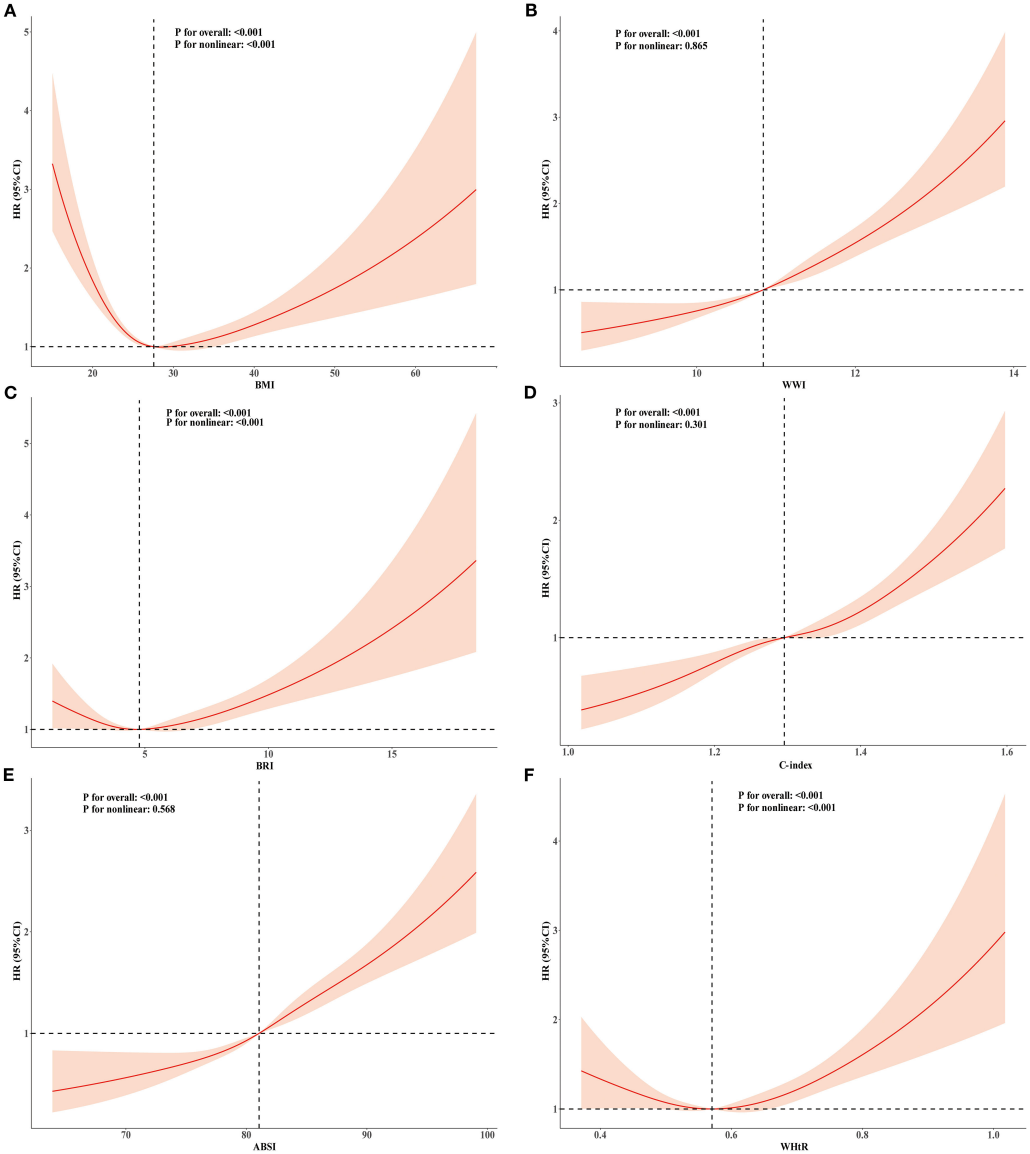
RCS analysis of the relationship between BMI, ABSI, WWI, C-index, BRI, WHtR and all-cause mortality in the population with CKM syndrome stages 0-3. Adjusted for age, sex, race, education level, marital status, uric acid, creatinine, blood urea nitrogen, FBG, HDL-C, LDL-C, TG, smoking, alcohol consumption, diabetes, and history of hypertension. **(A)** BMI; **(B)** WWI; **(C)** BRI; **(D)** C-index; **(E)** ABSI; **(F)** WHtR.

**Figure 3:**
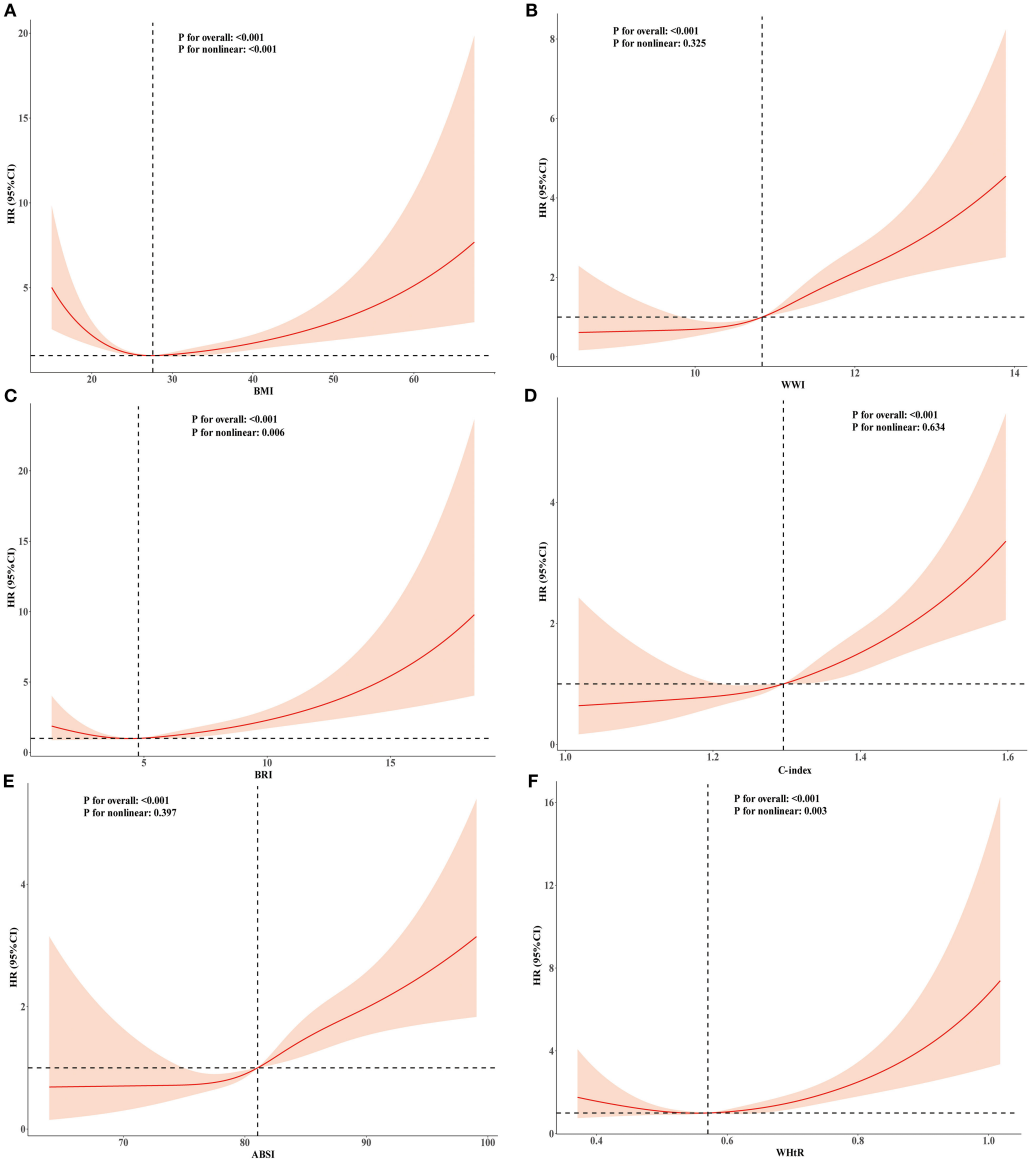
RCS analysis of the relationship between BMI, ABSI, WWI, C-index, BRI, WHtR and cardiovascular mortality in the population with CKM syndrome stages 0-3. Adjusted for age, sex, race, education level, marital status, uric acid, creatinine, blood urea nitrogen, FBG, HDL-C, LDL-C, TG, smoking,alcohol consumption, diabetes, and history of hypertension. **(A)** BMI; **(B)** WWI; **(C)** BRI; **(D)** C-index; **(E)** ABSI; **(F)** WHtR.

To explore the nonlinear relationships among BMI, novel obesity markers, and the risks of all-cause and cardiovascular mortality in patients with CKM, restricted cubic splines (RCS) were used in multivariate Cox regression models for evaluation. Our study revealed that after adjusting for potential confounding factors, continuous-scale BMI, BRI, and WHtR all demonstrated U-shaped associations with the risk of all-cause mortality. Both higher and lower BMI, BRI, and WHtR were associated with increased mortality risk (P-overall < 0.001, P-nonlinear < 0.001). For details, refer to **Figure 2**. In contrast, the ABSI, WWI, and C-index were linearly correlated with the risk of all-cause mortality (all P values > 0.05). Elevated ABSI, WWI, and C-index values were associated with an increased risk of mortality in CKM patients (all P values overall < 0.001). Similarly, in terms of the risk of cardiovascular mortality, BMI, BRI, and WHtR all demonstrated U-shaped associations (P-overall < 0.001, P-nonlinear < 0.001). ABSI, WWI, and C-index values also exhibited linear correlations with the risk of cardiovascular mortality. For details, please refer to **Figure 3**. To gain deeper insights into the dose–response relationship linking BRI and WHtR with the risk of mortality, a recursive method was utilized to identify possible inflection points. Additionally, a piecewise Cox proportional hazards model was adopted to elucidate the relationships on either side of these inflection points. We calculated the inflection point for the BRI to be 4.89 (log-likelihood ratio all P < 0.05). When the BRI score was less than 4.89, for every unit increase in the BRI score, there was a corresponding 14% decrease in the risk of all-cause mortality (HR: 0.86, 95% CI 0.79, 0.93). In contrast, when BRI levels surpassed 4.89, each unit increase correlated with a 6% increase in the risk of all-cause mortality (HR: 1.06, 95% CI 1.03, 1.09). The inflection point for WHtR*10 is 5.76. When the WHtR*10 level exceeds 5.76, each unit increase is associated with a 26% reduction in the risk of all-cause mortality (HR: 0.74, 95% CI 0.64, 0.86). Conversely, when the WHtR*10 is less than 5.76, each unit increase is associated with a 16% increase in the risk of all-cause mortality (HR: 1.16, 95% CI 1.07, 1.26). The details are shown in **Table 4**. With respect to cardiovascular mortality outcomes, no threshold effects were observed in the associations between WHtR or BRI and the outcomes (all log-likelihood ratios >0.05), as shown in **Supplementary Table S8**.

**Table 4:** Analysis of the threshold effect of BRI and WHtR on all-cause in patients with CKM syndrome stage 0–3.

All-cause mortality	Adjusted HR (95% CI)	*P*
BRI		
Fitting model by standard linear regression	1.01 (0.99 - 1.03)	0.491
Fitting model by two-piecewise linear regression		
Inflection point	4.89	
BRI <4.89	0.86 (0.79 - 0.93)	**<0.001**
BRI ≥4.89	1.06 (1.03 - 1.09)	**<0.001**
P for likelihood test		**<0.001**
WHtR*10		
Fitting model by standard linear regression	1.00 (0.96 - 1.06)	0.852
Fitting model by two-piecewise linear regression		
Inflection point	5.762	
<5.762	0.74 (0.64 - 0.86)	**<0.001**
≥5.762	1.16 (1.07 - 1.26)	**<0.001**
P for likelihood test		**<0.001**

Adjusted for age, sex, race, marital status, education, uric acid, serum creatinine, BUN, HDL-C, LDL-C, TG, FBG, HbA1c, smoking, drinking, hypertension, diabetes.

HR, hazards ratio; CI, confidence interval. Other abbreviations, see **Table 1**.

Bold font indicates statistically significant differences (P < 0.05).

### Subgroup analysis and sensitivity analyses of the associations between novel obesity markers and mortality

Analyses of subgroups and interactions were performed considering factors such as age, sex, smoking habits, alcohol intake, history of hypertension, diabetes, and stages of CKM syndrome (from stage 0 to stage 3) to evaluate whether baseline or demographic characteristics influenced the impact of new obesity markers on overall mortality. After adjusting for covariates, BRI, WWI, ABSI, C-index, and WHtR showed significant interactions with age (all P values for interactions < 0.05). The increase in mortality risk was greater in the 20–59 years age group than in the 60+ years age group. The associations between novel obesity markers and mortality in other distinct characteristic subgroups are detailed in **Table 5** and **Supplementary Materials Tables S11**-**S13**. In sensitivity analyses excluding participants who died within the first two years, results remained consistent(**Supplementary Tables S15**, **S16**). Similar findings were observed using the Fine-Gray proportional subdistribution hazards model (**Supplementary Table S14**).

**Table 5:** Subgroup analysis of the association between ABSI and all-cause and cardiovascular mortality in patients with CKM syndrome stage 0–3.

ABSI per 5 higher	All-cause mortality			Cardiovascular mortality		
	HR (95%CI)	*P*	*P* interaction	HR (95%CI)	*P*	*P* interaction
Age			**0.047**			**0.040**
<60 years	1.30 (1.19 ~ 1.42)	**<0.001**		1.70 (1.30 ~ 2.23)	**<0.001**	
≥ 60 years	1.28 (1.19 ~ 1.37)	**<0.001**		1.26 (1.10 ~ 1.44)	**0.001**	
Gender			0.167			0.342
Male	1.40 (1.26 ~ 1.55)	**<0.001**		1.46 (1.25 ~ 1.70)	**<0.001**	
Female	1.26 (1.16 ~ 1.37)	**<0.001**		1.28 (1.09 ~ 1.52)	**0.003**	
Smoking			0.189			0.169
Yes	1.32 (1.17 ~ 1.48)	**<0.001**		1.78 (1.38 ~ 2.28)	**<0.001**	
No	1.31 (1.23 ~ 1.38)	**<0.001**		1.30 (1.14 ~ 1.47)	**<0.001**	
Drinking			0.250			0.461
Yes	1.32 (1.23 ~ 1.42)	**<0.001**		1.53 (1.23 ~ 1.89)	**<0.001**	
No	1.30 (1.18 ~ 1.44)	**<0.001**		1.35 (1.17 ~ 1.55)	**<0.001**	
Diabetes			0.137			0.448
Yes	1.33 (1.25 ~ 1.41)	**<0.001**		1.37 (1.21 ~ 1.54)	**<0.001**	
No	1.25 (1.06 ~ 1.47)	**0.009**		1.34 (0.97 ~ 1.85)	0.079	
Hypertension			**0.005**			0.067
Yes	1.37 (1.26 ~ 1.48)	**<0.001**		1.47 (1.21 ~ 1.79)	**<0.001**	
No	1.27 (1.17 ~ 1.36)	**<0.001**		1.33 (1.15 ~ 1.54)	**<0.001**	
CKM			0.815			0.228
Stage 0	1.18 (0.78 ~ 1.79)	0.427		0.59 (0.20 ~ 1.72)	0.330	
Stage 1	1.20 (1.00 ~ 1.44)	**0.046**		1.02 (0.68 ~ 1.53)	0.918	
Stage 2	1.33 (1.24 ~ 1.42)	**<0.001**		1.42 (1.25 ~ 1.62)	**<0.001**	
Stage 3	1.32 (1.12 ~ 1.56)	**0.001**		1.41 (1.04 ~ 1.91)	**0.028**	

HR, hazard ratio; CI, confidence interval.

The model was adjusted for age, sex, race, marital status, education, uric acid, serum creatinine, BUN, HDL-C, LDL-C, TG, FBG, HbA1c, smoking, drinking, hypertension, diabetes.

Bold font indicates statistically significant differences (P < 0.05).

### Comparative analysis of the prediction of mortality

ROC curve analysis was used to assess the ability of the novel adiposity indices and BMI to predict the risk of all-cause mortality (**Supplementary Figure S1**). Among these indices, the ABSI had the highest AUC for all-cause mortality (0.717, 95%CI:0.706-0.727), followed by the C-index (0.670, 95%CI:0.659-0.680), WWI (0.666, 95%CI:0.655-0.676), WHtR (0.558, 95%CI:0.547-0.569), BRI (0.551, 95%CI:0.539-0.564), and BMI (0.532, 95%CI:0.521-0.543). Comparable findings were noted for the AUC concerning cardiovascular mortality (**Supplementary Figure S2**). After adding the novel obesity index to Model 3, the results indicated that WWI, ABSI, and C-index significantly improved the predictive performance for adverse outcomes. For predicting all-cause mortality, the continuous NRI values were 0.1831 (95% CI: 0.1289–0.1992), 0.2191 (0.1644–0.2877), and 0.1805 (0.1173–0.2398), with corresponding IDI values of 0.0356 (0.0193–0.0569), 0.0572 (0.0365–0.0839), and 0.0245 (0.0118–0.0432). Similar results were observed in terms of cardiovascular mortality, as shown in S17.

## Discussion

In this extensive cohort analysis, utilizing NHANES data from 2001-2018, we investigated the correlation between novel obesity indices and mortality in the CKM syndrome cohort. Our findings indicate that (1) there is a significant positive correlation between novel obesity indices and all-cause and cardiovascular mortality, and (2) the obesity paradox related to BMI persists in the CKM syndrome cohort; individuals with overweight/obesity presented a reduced all-cause mortality risk compared with their normal-weight counterparts. (3) Significant nonlinear correlations were observed between BMI, WHtR, and BRI and all-cause mortality as well as cardiovascular mortality. (4) Significant positive linear correlations were found between the WWI, ABSI, and C-index and between all-cause mortality and cardiovascular mortality. (5) Increases in the WWI, ABSI, C-index, BRI, and WHtR were associated with greater increases in all-cause mortality risk among individuals aged 20–59 years than among those aged 60 years or older. (6) New obesity markers were superior to BMI in predicting both cardiovascular mortality and all-cause mortality.

The convergence of metabolic risk factors and CKD in CKM syndrome patients is closely associated with the risk of cardiovascular morbidity and adverse outcomes. The most common etiology of CKM syndrome is excessive or dysfunctional adipose tissue, particularly visceral adipose tissue, which can secrete proinflammatory and pro-oxidative products that damage arterial, cardiac, and renal tissues. Simultaneously, inflammatory processes reduce sensitivity to insulin, leading to impaired glucose tolerance and subsequently triggering abnormalities in the metabolic, cardiac, and renal dimensions (11, 12, 26, 27). Therefore, obesity plays a crucial role in the development and prognosis of CKM syndrome. In this study, we found a significant positive correlation between novel obesity indices and all-cause mortality as well as cardiovascular mortality. Traditionally, BMI has been used to define obesity, but it has limitations in reflecting obesity management and predicting adverse outcomes. BMI is unable to distinguish among the various elements that make up body composition, including obesity, lean mass, and skeletal muscle. It also does not differentiate between different distributions of fat, such as central and peripheral fat deposition, nor does it account for factors such as fluid retention in cases of decompensated heart failure (28). In response to the limitations of the BMI index, this study introduces innovative obesity indicators such as the ABSI, WWI, BRI, C-index, and BRI. These metrics originate from waist circumference and are modified according to height and/or weight. WC serves as an anthropometric assessment of central obesity and proves to be more efficient than BMI in detecting cardiometabolic conditions (29). A nationwide cohort study in South Korea demonstrated that WWI has good predictive value for the incidence and mortality of cardiometabolic diseases (30). A cohort study conducted on Iranian adults demonstrated a statistically significant association between ABSI and all-cause mortality, indicating that ABSI can serve as an independent predictor of mortality risk (31). A cohort study conducted in China on the elderly population revealed that the C-index can serve as an independent risk factor for assessing mortality risk in this demographic population (32). The results of a nationwide prospective cohort study on Chinese adults suggest that the BRI can enhance the ability of the BRI to predict CVD mortality and cardiovascular death risk (33).

Research has indicated that, in contrast to those with a healthy weight, individuals classified as overweight or obese (according to traditional BMI standards) have a decreased risk for both all-cause and cardiovascular disease-related death (14, 34). This phenomenon is often termed the obesity paradox. In our study, we found that, compared with patients with a normal weight, overweight and obese patients had a lower risk of all-cause mortality, which is consistent with previous research findings. This finding indicates that in the early CKM population, BMI, as an indicator for assessing obesity, still presents an obesity paradox. Prior research has identified lower BMI as an independent predictor of mortality risk in heart failure patients, but mortality increases once BMI exceeds 45 kg/m², suggesting a nonlinear parabolic relationship between BMI and mortality risk (U-shaped), with both extremes of adiposity demonstrating elevated hazard ratios (35). Our study also confirmed the U-shaped association between BMI and mortality. An increase in BMI affects left ventricular remodeling, and a higher BMI contributes to augmented cardiac mechanical loading, which may lead to adverse outcomes (36, 37).

As previously mentioned, when considered categorical variables, after adjusting for potential covariates, patients with higher BMIs were associated with lower mortality risk, whereas higher levels of the novel obesity marker corresponded to increased mortality risk. When BMI was treated as a continuous variable, it was not significantly associated with mortality risk, whereas the novel obesity marker consistently demonstrated associations with mortality risk. These findings suggest that the novel obesity index may better represent body fat distribution and offer advantages in managing obesity and predicting adverse outcomes. Furthermore, our findings revealed distinct obesity–mortality associations: the WWI, ABSI, and C-index were linearly related to both all-cause mortality and cardiovascular mortality, whereas the WHtR and BRI were nonlinearly U-shaped. NHANES data analysis revealed a U-shaped correlation between the BRI index and all-cause mortality as well as cardiovascular issues in individuals diagnosed with diabetes (38). A cohort study of individuals with metabolic syndrome also demonstrated a U-shaped association between the BRI index and mortality (all-cause/cardiovascular) (39). Research involving populations experiencing hyperlipidemia revealed a U-shaped relationship between WHtR and both overall mortality and cardiovascular-related deaths (40). Therefore, given the nonlinear relationship between WHtR and BRI with mortality risk, a recursive approach was used to detect possible inflection points, and a piecewise Cox regression was applied to describe the relationships on both sides of these identified inflection points. The results indicated that above and below different inflection points, the corresponding outcomes were opposite, indicating that both elevated and lowered BRI and WHtR are linked to the risk of mortality in individuals with CKM syndrome and that keeping these metrics within a desirable range could lower mortality rates. The inflection points identified in our study may provide a reference for obesity management standards in CKM patients.

According to the subgroup analyses and interaction tests, age had significant interaction effects on the ABSI, C-index, BRI, WHtR, and WWI, with individuals aged 20–59 years exhibiting a greater increase in mortality risk than those aged 60 years and older. We speculate the following reasons. Firstly, elderly individuals often experience visceral fat accumulation and muscle loss (sarcopenic obesity) (41). Some current obesity markers may fail to distinguish between low muscle mass and high fat mass, leading to an underestimation of mortality risk. In addition, the secretory profile and metabolic activity of adipose tissue in elderly obese patients are altered, and the proinflammatory factors secreted by adipose tissue in elderly obese individuals may be partially offset by age-related immune senescence (42). In the elderly population, nonmetabolic causes of death, such as dementia and cancer, account for a greater proportion of deaths, diluting the independent risk of obesity indicators themselves. A previous multicenter prospective cohort study demonstrated that in the elderly population aged 80 years and above, elevated BMI levels were independently and inversely associated with the risk of all-cause mortality (43). Notably, although the increase in mortality risk associated with novel obesity markers is lower in elderly individuals than in younger individuals, unlike BMI, these new markers still maintain a positive correlation with mortality risk. Therefore, compared with BMI, these new obesity markers may serve as better indicators for the assessment and management of obesity in the elderly population. The ABSI, C-index, BRI, and WHtR showed more significant associations with mortality risk in CKM stage 2, whereas the WWI demonstrated a notable increase in mortality risk in CKM stages 2–3, with the increase being nearly equivalent. These findings suggest that new obesity indicators may improve the predictive accuracy of all-cause mortality. in individuals with CKD who have metabolic risk factors. However, the results of our study suggest that there was no statistically significant interaction among the various groups of CKM syndrome stages 0-3. This highlights the necessity for more comprehensive prospective cohort studies to determine whether the relationship between novel obesity markers and mortality risk remains consistent throughout all CKM syndrome stages.

CKM syndrome is a common, chronic disease affecting multiple systems in the body. Research indicates that this condition arises from either excessive or dysfunctional obesity, leading to systemic inflammation and oxidative stress (12). In cases of obesity, proinflammatory factors that are overproduced, such as prototypic inflammatory markers (TNF-α, IL-6, and MCP-1), have the potential to trigger local inflammation and oxidative stress within various tissues and organs. This process can subsequently indirectly contribute to cardiovascular dysfunction and metabolic disorders (12). Conversely, the adipose tissue of lean individuals primarily produces anti-inflammatory molecules, such as transforming growth factor-β, interleukin-4, interleukin-10, and interleukin-13 (44). In cases of severe obesity, the elevation of lipocalin is attenuated, resulting in decreased anti-inflammatory and atherosclerosis-inhibiting capacities (45). In addition, adipocytes release aldosterone or generate factors stimulating aldosterone secretion, which activate adrenal glands to promote excess aldosterone production, thereby leading to overactivation of MR. This, in turn, induces glomerular and interstitial fibrosis in the kidneys through oxidative stress and inflammatory responses (46, 47). Therefore, obesity is closely associated with the development and adverse outcomes of cardiovascular, metabolic, and renal diseases. This study revealed that, compared with the traditional obesity indicator BMI, the new obesity markers WWI, ABSI and C-index, have greater advantages in assessing obesity levels and predicting adverse outcomes in the early-stage CKM syndrome population. They can serve as effective alternative indicators for recognizing the risk of adverse outcomes.

Nonetheless, the limitations of our research should not be ignored. First, because of the observational design, we are unable to determine causality. Second, the information for this research was obtained from the NHANES, with the sample group reflecting mainly U.S. demographics, which may not be applicable globally. Third, instead of utilizing the most recent PREVENT equation to define subclinical cardiovascular disease, we chose to employ the Framingham equation to predict the 10-year cardiovascular risk score. Fourth, some variables were based on self-reported data, introducing the risk of recall bias and inaccuracies. Fifth, despite adjustments for multiple covariates and sensitivity analyses, potential confounding factors may still have been overlooked. Finally, the dynamic changes in obesity and the progression of CKM stages are difficult to capture, which may affect the relationship with outcomes in this prospective analysis.

## Conclusions

In conclusion, this study highlights the correlation between BMI, novel obesity indices, and mortality in patients with stages 0–3 CKM syndrome. The ABSI, C-index, BRI, WHtR, and WWI were positively correlated with all-cause mortality and cardiovascular death in individuals with stage early-stage CKM syndrome. The new obesity markers offer greater advantages in assessing obesity levels and predicting adverse outcomes in the early-stage CKM syndrome population. This finding identifies actionable targets for lifestyle improvement and weight management, supporting long-term health maintenance in high-risk populations with cardiorenal metabolic disorders.

## Data Availability

The original contributions presented in the study are included in the article/**Supplementary Material**, further inquiries can be directed to the corresponding author/s.
