# Supplementary material for: Novel adiposity indices and their associations with all-cause and cardiovascular mortality in individuals with cardiovascular–kidney–metabolic syndrome stages 0–3: findings from a nationwide prospective cohort study
Source: Front Endocrinol (Lausanne). 2025 Oct 7;16:1660210. doi: 10.3389/fendo.2025.1660210 (PMC12537431; doi:10.3389/fendo.2025.1660210)
Supplement: Supplementary file 1 [file Table1.docx]

Supplementary Material

# 1 Supplemental methods

**Details of the statistical analyses**.

This research utilized a sophisticated sampling design along with sample weights, following the protocols outlined by the National Health and Nutrition Examination Survey (NHANES) analysis. To calculate the sample weights, a specific method was employed: the weight for the fasting subsample at the mobile examination center (MEC) was derived by taking the fasting subsample two-year MEC weight and dividing it by the factor of nine. This approach ensures that the sampling design adheres to established standards, thus enhancing the validity of the findings.

Continuous variables following a normal distribution are expressed as weighted mean (standard error), continuous variables conforming to a normal distribution are expressed as median (interquartile range), and categorical variables are expressed as frequency (weighted percentage). Continuous variables following a normal distribution are analyzed using t-tests, while continuous variables not conforming to a normal distribution are analyzed using non-parametric rank-sum tests. Baseline characteristic differences of categorical variables are analyzed using survey-weighted chi-square tests. Univariate and multivariate Cox proportional hazards regression models were employed, with the novel obesity indices and BMI entered as both continuous and categorical variables, to calculate their hazard ratios (HRs) and 95% confidence intervals (CIs) for all-cause and cardiovascular mortality. Three regression models were constructed: Model 1: unadjusted; Model 2: adjusted for age, sex, and race; Model 3: adjusted for age, sex, race, education level, marital status, uric acid, creatinine, blood urea nitrogen, FBG, HDL-C, LDL-C, TG, smoking, alcohol consumption, diabetes, and history of hypertension. The proportional hazards assumption was examined using Schoenfeld residuals, with no evidence of violations observed. Considering the issue of collinearity, we examined the variance inflation factor (VIF) of the covariates to include only those with a VIF <5 in the model.Table S2-TableS6.

Restricted cubic spline (RCS) curves with 4 knots(at the 5th, 35th, 65th.and 95th percentiles) were used to assess the dose-response relationships between BRI, WHtR, WWI, C-index, ABSI, BMI and the endpoints, including all-cause mortality and cardiovascular mortality. In the case of nonlinear relationships, we employ a recursive approach to identify potential inflection points and utilize a piecewise Cox proportional hazards model to characterize the associations on both sides of the inflection points.

Sensitivity analyses were conducted to verify the robustness of the results. To investigate potential reverse causality, participants who died within the first 2 years of follow-up were excluded. Re-ran all Cox regression models on the remaining cohort.Secondly, Fine-Gray proportional subdistribution hazard models were employed to assess the associations of the novel obesity index and BMI with cardiovascular mortality outcomes, with non-cardiovascular deaths treated as competing risk events. Stratified analyses examined effect modifications by demographic and clinical variables, including age, sex, lifestyle factors (alcohol consumption/smoking), cardiovascular and metabolic diseases, and CKM stages, with likelihood ratio tests used to evaluate subgroup interactions.

ROC analyses with area under the curve (AUC) quantification were performed to assess the predictive performance of novel adiposity metrics versus conventional BMI for mortality outcomes. The predictive performance of these novel metrics and BMI for adverse outcomes was compared using continuous net reclassification improvement (NRI) and integrated discrimination improvement (IDI).

**NHANES-NDI Mortality Linkage Methodology**

Mortality data from the National Health and Nutrition Examination Survey (NHANES) were linked to the National Death Index (NDI) through a rigorous hybrid methodology:

1.**Deterministic Linkage**:

Records with full (SSN9) or partial (SSN4) Social Security Numbers were matched *exactly* to NDI using SSN.

Validation required ≥50% agreement on non-missing identifiers (name, birth date, sex) for SSN9 matches; ≥65% for SSN4.

2.**Probabilistic Linkage**:

For unmatched records, candidate pairs were scored using the **Fellegi-Sunter model**.

Agreement weights (*Ai​)* and disagreement weights (*Di​)* for identifiers (name, birth date, etc.) were computed as:

*A_i_*=log_2_(*m_i_/u_i_*), *D_i_*=log_2_(1−*m_i_/*1−*u_i_*)

Where *m_i_*= probability of agreement if a true match, *u_i_*_​_= random agreement probability.

**Jaro-Winkler similarity** resolved name variations (e.g., nicknames, typos).

Match probabilities were estimated via a partial **Expectation-Maximization algorithm**.

3.**Match Selection**:

Pairs with a match probability ≥ **0.99** were accepted.

Type I (false positive) and Type II (false negative) errors were minimized to **<2%**.

4.**NHANES-Specific Handling**:

Follow-up time was calculated in person-months from interview/exam date to death or December 31, 2019.

Underlying causes of death were classified using **ICD-9 (pre-1999)** or **ICD-10 (1999–2019)**, aggregated into 113 recode groups.

Public-use files applied data perturbation (synthetic dates/causes) to protect confidentiality; vital status remained unaltered

# 2 Supplemental tables

**Table S1 Definition of CKM**[1]

| **CKM health stage** | **CKM health stage** |
| --- | --- |
| Stage 0: No CKM  health risk factors | Individuals without overweight/obesity, metabolic risk factors (hypertriglyceridemia, hypertension, diabetes, MeTS), CKD or subclinical/clinical CVD  (1)BMI between 18.5 and 25 kg/m^2^, inclusive  (2)WC <102 cm for men or <88 cm for women |
| Stage 1: Excess  and/or dysfunctional  adiposity | Individuals with overweight/obesity, abdominal obesity, or adipose tissue dysfunction without other metabolic risk factors, CKD, or subclinical/clinical CVD  (1)BMI ≥ 25 kg/m^2^  (2)WC ≥ 102 cm for men or ≥ 88 cm for women  (3)FBG levels ranging from 100 to 124 mg/dL, or HbA1c levels between 5.7 and 6.4 %* |
| Stage 2: ­Metabolic  risk factors and  CKD | Individuals with metabolic risk factors (hypertriglyceridemia, hypertension, diabetes, †MeTS) or moderate to high-risk CKD stage(The stage of CKD is determined by the KDIGO criteria[2], using eGFR and UACR. The eGFR was calculated using the 2021 race and ethnicity‐free Chronic Kidney Disease Epidemiology Collaboration creatinine equation[3]).  (1)TG >135 mg/dL  (2)Hypertension is defined by an SBP of ≥130 mm Hg, a DBP of ≥80 mm Hg, a medical diagnosis, or taking antihypertensive medication.  (3)Diabetes is defined by FBG levels of > 126 mg/dL, HbA1c levels of ≥ 6.5%, a medical diagnosis, or taking insulin or glucose-lowering medication.  (4) Moderate to high-risk CKD in the KDIGO classification is defined as UACR ≥ 30 mg/g and eGFR ≥ 60 ml/min/1.73m^2^, UACR < 300 mg/g and eGFR ≤ 45-59 ml/min/1.73m^2^, or UACR < 30 mg/g and eGFR ≤ 30-44 ml/min/1.73m^2^. |
| Stage 3: Subclinical  CVD in CKM | Risk equivalents for subclinical CVD: high predicted 10-year CVD risk or very high-risk KDIGO CKD stage  (1)A high 10-year CVD risk is defined as a 20% or above risk, as determined by the basic Predicting Risk of CVD EVENTs (Framingham) equation[4].  (2)Very high-risk CKD in the KDIGO classification is defined as UACR ≥ 300 mg/g and eGFR ≤ 45-59 ml/min/1.73 m^2^, UACR ≥ 30 mg/g and eGFR ≤ 30-44 ml/min/1.73 m^2^, or eGFR ≤ 29 ml/min/1.73 m^2^. |
| Stage 4: Clinical  CVD in CKM | Clinical CVD (self-reported diagnosed cardiovascular disease, including heart failure, coronary heart disease, angina, heart attack, and stroke) in individuals |

AFib indicates atrial fibrillation; ASCVD, atherosclerotic cardiovascular disease; BMI, body mass index; CKD, chronic kidney disease; CKM, cardiovascular-kidney-metabolic; CT, computed tomography; CVD, cardiovascular disease; HbA1c, hemoglobin A1c; HF, heart failure; KDIGO, Kidney Disease Improving Global Outcomes; MetS, metabolic syndrome; and NT-proBNP, N-terminal pro-B-type natriuretic peptide. *Individuals with gestational diabetes should receive intensified screening for impaired glucose tolerance after pregnancy.

**†**MetS is defined by the presence of ≥3 of the following: (1) waist circumference ≥88 cm for women and ≥102 cm for men (if Asian ancestry, ≥80 cm for women and ≥90 cm for men), (2) high-density cholesterol <40 mg/dL for men and <50 mg/dL for women; (3) triglycerides ≥150 mg/dL; (4) elevated blood pressure (systolic blood pressure ≥130 mm Hg and/or diastolic blood pressure ≥80 mm Hg and/or use of antihypertensive medications); and (5) fasting blood glucose ≥100 mg/dL.

**Table S2**. Collinearity diagnostics steps involving BRI.

|  | VIF |
| --- | --- |
| BRI | 1.1 |
| Sex | 1.6 |
| Age | 1.9 |
| Race | 1.1 |
| Education | 1.1 |
| Marita | 1.1 |
| Diabetes | 1.6 |
| Hypertension | 1.4 |
| Smoking status | 1.1 |
| Drinking status | 1.1 |
| Systolic blood pressure | 1.6 |
| Diastolic blood pressure | 1.2 |
| Scr | 1.5 |
| Triglyceride | 1.3 |
| HDL-C | 1.5 |
| LDL-C | 1.2 |
| Fasting plasma glucose | 2.7 |
| HbA1c | 3.2 |
| BUN | 1.5 |
| Uric acid, | 1.6 |
| CKM syndrome | 1.3 |

**Table S3**. Collinearity diagnostics steps involving WWI.

|  | VIF |
| --- | --- |
| WWI | 1.3 |
| Sex | 1.7 |
| Age | 2.3 |
| Race | 1.1 |
| Education | 1.1 |
| Marita | 1.1 |
| Diabetes | 1.6 |
| Hypertension | 1.4 |
| Smoking status | 1.1 |
| Drinking status | 1.1 |
| Systolic blood pressure | 1.6 |
| Diastolic blood pressure | 1.3 |
| Scr | 1.5 |
| Triglyceride | 1.3 |
| HDL-C | 1.5 |
| LDL-C | 1.2 |
| Fasting plasma glucose | 2.7 |
| HbA1c | 3.2 |
| BUN | 1.5 |
| Uric acid, | 1.6 |
| CKM syndrome | 1.3 |

**Table S4**. Collinearity diagnostics steps involving WHtR.

|  | VIF |
| --- | --- |
| WHtR | 1.5 |
| Sex | 1.6 |
| Age | 1.9 |
| Race | 1.1 |
| Education | 1.1 |
| Marita | 1.1 |
| Diabetes | 1.6 |
| Hypertension | 1.4 |
| Smoking status | 1.1 |
| Drinking status | 1.1 |
| Systolic blood pressure | 1.6 |
| Diastolic blood pressure | 1.2 |
| Scr | 1.5 |
| Triglyceride | 1.3 |
| HDL-C | 1.5 |
| LDL-C | 1.2 |
| Fasting plasma glucose | 2.7 |
| HbA1c | 3.2 |
| BUN | 1.5 |
| Uric acid, | 1.5 |
| CKM syndrome | 1.3 |

**Table S5**. Collinearity diagnostics steps involving ABSI.

|  | VIF |
| --- | --- |
| ABSI | 1.4 |
| Sex | 1.6 |
| Age | 2.2 |
| Race | 1.1 |
| Education | 1.1 |
| Marita | 1.1 |
| Diabetes | 1.6 |
| Hypertension | 1.4 |
| Smoking status | 1.1 |
| Drinking status | 1.1 |
| Systolic blood pressure | 1.6 |
| Diastolic blood pressure | 1.2 |
| Scr | 1.5 |
| Triglyceride | 1.3 |
| HDL-C | 1.5 |
| LDL-C | 1.2 |
| Fasting plasma glucose | 2.7 |
| HbA1c | 3.2 |
| BUN | 1.5 |
| Uric acid, | 1.6 |
| CKM syndrome | 1.3 |

**Table S6**. Collinearity diagnostics steps involving C-index.

|  | VIF |
| --- | --- |
| C-index | 1.4 |
| Sex | 1.6 |
| Age | 1.9 |
| Race | 1.1 |
| Education | 1.1 |
| Marita | 1.1 |
| Diabetes | 1.6 |
| Hypertension | 1.3 |
| Smoking status | 1.1 |
| Drinking status | 1.1 |
| Systolic blood pressure | 1.2 |
| Diastolic blood pressure | 1.6 |
| Scr | 1.5 |
| Triglyceride | 1.6 |
| HDL-C | 1.6 |
| LDL-C | 1.7 |
| Fasting plasma glucose | 2.7 |
| HbA1c | 3.3 |
| BUN | 1.5 |
| Uric acid, | 1.6 |
| CKM syndrome | 1.3 |

VIF = 1/(1-R^2^). VIF step-by-step screening method: Calculate the VIF of each variable. If the maximum VIF value is≥5, remove the variable with the maximum VIF value.

VIF: variance inflation factors. For other abbreviations, see Table 1.

**TableS7** Associations between the BMI and study outcome.

| Variable | Model 1  HR (95% CI) | *P* value | Model 2  HR (95% CI) | *P* value | Model3  HR (95% CI) | *P* value |
| --- | --- | --- | --- | --- | --- | --- |
| **All-cause mortality** |  |  |  |  |  |  |
| Per 1 higher | 1.01 (1.00 - 1.01) | 0.196 | 1.00 (0.99 - 1.01) | 0.806 | 0.99 (0.98 - 1.01) | 0.380 |
| BMI＜18.5kg/m^2^ | 1.00 (Reference) |  | 1.00 (Reference) |  | 1.00 (Reference) |  |
| 18.5≤BMI＜25kg/m^2^ | 0.54 (0.41 - 0.72) | **<0.001** | 0.44 (0.33 - 0.59) | **<0.001** | 0.55 (0.40 - 0.73) | **<0.001** |
| 25≤BMI＜30kg/m^2^ | 0.57 (0.43 - 0.76) | **<0.001** | 0.36 (0.27 - 0.48) | **<0.001** | 0.44 (0.32 - 0.60) | **<0.001** |
| BMI≥30kg/m^2^ | 0.61 (0.45 - 0.81) | **<0.001** | 0.42 (0.32 - 0.56) | **<0.001** | 0.46 (0.34 - 0.62) | **<0.001** |
| **Cardiovascular mortality** |  |  |  |  |  |  |
| Per 1 higher | 1.03 (1.01 - 1.04) | **0.004** | 1.03 (1.01 - 1.05) | **0.022** | 1.02 (0.99 - 1.04) | 0.237 |
| BMI＜18.5kg/m^2^ | 1.00 (Reference |  | 1.00 (Reference |  | 1.00 (Reference |  |
| 18.5≤BMI＜25kg/m^2^ | 0.63 (0.30 - 1.33) | 0.227 | 0.50 (0.25 - 1.03) | 0.062 | 0.59 (0.28 - 1.23) | 0.157 |
| 25≤BMI＜30kg/m^2^ | 0.75 (0.38 - 1.45) | 0.391 | 0.43 (0.23 - 0.82) | **0.010** | 0.49 (0.25 - 0.95) | **0.035** |
| BMI≥30kg/m^2^ | 0.92 (0.46 - 1.84) | 0.822 | 0.61 (0.32 - 1.16) | 0.130 | 0.59 (0.30 - 1.17) | 0.130 |

Model 1: No adjustments

Model 2: Adjusted for age, sex, race

Model 3: Adjusted for age, sex, race, marital status, education, uric acid, serum creatinine, BUN, HDL-C, LDL-C, TG, FBG, HbA1c, smoking, drinking, hypertension, diabetes

Abbreviations: HR, hazards ratio; CI, confidence interval. Other abbreviations, see Table 1.

**Table S8**. Analysis of the threshold effect of BRI and WHtR on cardiovascular mortality in patients with CKM syndrome stage 0–3.

| All-cause mortality | Adjusted HR (95% CI) | *P* |
| --- | --- | --- |
|  |  |  |
| BRI |  |  |
| Fitting model by standard linear regression | 1.04 (1.00 - 1.09) | 0.060 |
| Fitting model by two-piecewise linear regression |  |  |
| Inflection point | 5.68 |  |
| <5.68 | 0.91 (0.80 - 1.04) | 0.167 |
| ≥5.68 | 1.10 (1.02 - 1.17) | **0.008** |
| P for likelihood test |  | 0.105 |
| WHtR*10 |  |  |
| Fitting model by standard linear regression | 1.09 (0.98 - 1.21) | 0.132 |
| Fitting model by two-piecewise linear regression |  |  |
| Inflection point | 6.921 |  |
| <6.921 | 0.94 (0.80 - 1.11) | 0.468 |
| ≥6.921 | 1.46 (1.04 - 2.04) | **0.030** |
| P for likelihood test |  | 0.057 |

Bold font indicates that the log likelihood ratio test is statistically significant

(P < 0.05)

Adjusted for age, sex, race,marital status, education, uric acid,serum creatinine, BUN, HDL-C, LDL-C, TG,FBG,HbA1c, smoking, drinking, hypertension, diabetes

Abbreviations: HR, hazards ratio; CI, confidence interval. Other abbreviations, see Table 1.

**Table S9** Subgroup analysis of the association between WWI and all-cause and cardiovascular mortality in patients with CKM syndrome stage 0–3

| WWI  per 1 higher | All-cause mortality | | | Cardiovascular mortality | | |
| --- | --- | --- | --- | --- | --- | --- |
|  | HR (95%CI) | *P* | *P* interaction | HR (95%CI) | *P* | *P* interaction |
| Age |  |  | **0.011** |  |  | **0.002** |
| <60 years | 1.37 (1.21 ~ 1.55) | **<0.001** |  | 2.19 (1.57 ~ 3.04) | **<0.001** |  |
| ≥ 60 years | 1.35 (1.24 ~ 1.47) | **<0.001** |  | 1.46 (1.20 ~ 1.77) | **<0.001** |  |
| Gender |  |  | 0.081 |  |  | 0.193 |
| Male | 1.53 (1.34 ~ 1.74) | **<0.001** |  | 1.84 (1.49 ~ 2.27) | **<0.001** |  |
| Female | 1.31 (1.17 ~ 1.46) | **<0.001** |  | 1.42 (1.08 ~ 1.85) | **0.011** |  |
| Smoking |  |  | 0.284 |  |  | 0.223 |
| Yes | 1.33 (1.14 ~ 1.54) | **<0.001** |  | 2.43 (1.70 ~ 3.47) | **<0.001** |  |
| No | 1.41 (1.31 ~ 1.52) | **<0.001** |  | 1.53 (1.28 ~ 1.84) | **<0.001** |  |
| Drinking |  |  | 0.082 |  |  | 0.461 |
| Yes | 1.45 (1.32 ~ 1.60) | **<0.001** |  | 1.59 (1.20 ~ 2.11) | **0.001** |  |
| No | 1.30 (1.14 ~ 1.50) | **<0.001** |  | 1.71 (1.41 ~ 2.08) | **<0.001** |  |
| Diabetes |  |  | 0.137 |  |  | 0.186 |
| Yes | 1.33 (1.25 ~ 1.41) | **<0.001** |  | 1.64 (1.36 ~ 1.98) | **<0.001** |  |
| No | 1.25 (1.06 ~ 1.47) | **0.009** |  | 1.39 (0.94 ~ 2.05) | 0.095 |  |
| Hypertension |  |  | 0.069 |  |  | 0.469 |
| Yes | 1.38 (1.25 ~ 1.52) | **<0.001** |  | 1.67 (1.24 ~ 2.26) | **<0.001** |  |
| No | 1.43 (1.27 ~ 1.60) | **<0.001** |  | 1.70 (1.38 ~ 2.10) | **<0.001** |  |
| CKM |  |  | 0.795 |  |  | 0.426 |
| Stage 0 | 1.09 (0.58 ~ 2.05) | 0.786 |  | 0.00 (0.01 ~ 0.02) | <0.001 |  |
| Stage 1 | 1.34 (1.02 ~ 1.75) | **0.034** |  | 1.35 (0.76 ~ 2.42) | 0.306 |  |
| Stage 2 | 1.41 (1.31 ~ 1.53) | **<0.001** |  | 1.67 (1.40 ~ 2.00) | **<0.001** |  |
| Stage 3 | 1.42 (1.17 ~ 1.72) | **<0.001** |  | 1.98 (1.21 ~ 3.25) | **0.007** |  |

Bold font indicates statistically significant differences (P < 0.05)

HR, hazard ratio; CI, confidence interval;

The model was adjusted for age, sex, race, marital status, education, uric acid, serum creatinine, BUN, HDL-C, LDL-C, TG, FBG, HbA1c, smoking, drinking, hypertension, diabetes

**Table S10** Subgroup analysis of the association between BRI and all-cause and cardiovascular mortality in patients with CKM syndrome stage 0–3

| BRI  per 1 higher | All-cause mortality | | | Cardiovascular mortality | | |
| --- | --- | --- | --- | --- | --- | --- |
|  | HR (95%CI) | *P* | *P* interaction | HR (95%CI) | *P* | *P* interaction |
| Age |  |  | **<0.001** |  |  | **0.002** |
| <60 years | 1.07 (1.02 ~ 1.12) | **0.007** |  | 1.19 (1.09 ~ 1.31) | **<0.001** |  |
| ≥ 60 years | 1.02 (0.98 ~ 1.05) | 0.352 |  | 1.08 (1.00 ~ 1.17) | **0.049** |  |
| Gender |  |  | **0.006** |  |  | **0.009** |
| Male | 1.10 (1.05 ~ 1.15) | **<0.001** |  | 1.20 (1.11 ~ 1.30) | **<0.001** |  |
| Female | 1.00 (0.96 ~ 1.05) | 0.861 |  | 1.03 (0.94 ~ 1.13) | 0.567 |  |
| Smoking |  |  | 0.585 |  |  | 0.087 |
| Yes | 1.04 (0.99 ~ 1.10) | 0.154 |  | 1.27 (1.14 ~ 1.41) | **<0.001** |  |
| No | 1.05 (1.01 ~ 1.08) | **0.005** |  | 1.09 (1.01 ~ 1.18) | **0.020** |  |
| Drinking |  |  | **0.020** |  |  | 0.131 |
| Yes | 1.00 (0.95 ~ 1.06) | 0.857 |  | 1.05 (0.96 ~ 1.15) | 0.273 |  |
| No | 1.07 (1.03 ~ 1.11) | **<.001** |  | 1.16 (1.08 ~ 1.25) | **<0.001** |  |
| Diabetes |  |  | 0.983 |  |  | 0.073 |
| Yes | 1.04 (0.97 ~ 1.12) | 0.290 |  | 0.98 (0.87 ~ 1.11) | 0.764 |  |
| No | 1.05 (1.01 ~ 1.08) | **0.005** |  | 1.14 (1.06 ~ 1.23) | **<0.001** |  |
| Hypertension |  |  | 0.648 |  |  | 0.459 |
| Yes | 1.05 (1.01 ~ 1.09) | **0.018** |  | 1.15 (1.06 ~ 1.24) | **<0.001** |  |
| No | 1.04 (1.00 ~ 1.09) | 0.055 |  | 1.09 (1.00 ~ 1.20) | 0.059 |  |
| CKM |  |  | 0.734 |  |  | 0.103 |
| Stage 0 | 1.09 (0.66 ~ 1.79) | 0.734 |  | 0.00 (0.01 ~ 0.01) | <0.001 |  |
| Stage 1 | 1.05 (0.95 ~ 1.16) | 0.317 |  | 1.07 (0.91 ~ 1.27) | 0.424 |  |
| Stage 2 | 1.05 (1.01 ~ 1.08) | **0.006** |  | 1.13 (1.05 ~ 1.22) | **0.001** |  |
| Stage 3 | 1.03 (0.96 ~ 1.10) | 0.379 |  | 1.13 (0.99 ~ 1.29) | 0.079 |  |

Bold font indicates statistically significant differences (P < 0.05)

HR, hazard ratio; CI, confidence interval;

The model was adjusted for age, sex, race, marital status, education, uric acid, serum creatinine, BUN, HDL-C, LDL-C, TG, FBG, HbA1c, smoking, drinking, hypertension, diabetes

**Table S11** Subgroup analysis of the association between C-index and all-cause and cardiovascular mortality in patients with CKM syndrome stage 0–3

| C-index  per 0.1 higher | All-cause mortality | | | Cardiovascular mortality | | |
| --- | --- | --- | --- | --- | --- | --- |
|  | HR (95%CI) | *P* | *P* interaction | HR (95%CI) | *P* | *P* interaction |
| Age |  |  | **0.002** |  |  | **0.008** |
| <60 years | 1.28 (1.16 ~ 1.42) | **<0.001** |  | 1.84 (1.38 ~ 2.45) | **<0.001** |  |
| ≥ 60 years | 1.21 (1.12 ~ 1.31) | **<0.001** |  | 1.26 (1.07 ~ 1.49) | **0.007** |  |
| Gender |  |  | 0.071 |  |  | 0.089 |
| Male | 1.38 (1.23 ~ 1.55) | **<0.001** |  | 1.61 (1.32 ~ 1.95) | **<0.001** |  |
| Female | 1.19 (1.07 ~ 1.32) | **0.001** |  | 1.21 (0.96 ~ 1.52) | 0.109 |  |
| Smoking |  |  | 0.467 |  |  | 0.056 |
| Yes | 1.25 (1.10 ~ 1.42) | **<0.001** |  | 2.18 (1.59 ~ 2.98) | **<0.001** |  |
| No | 1.27 (1.19 ~ 1.36) | **<0.001** |  | 1.31 (1.12 ~ 1.53) | **<0.001** |  |
| Drinking |  |  | 0.066 |  |  | 0.882 |
| Yes | 1.20 (1.07 ~ 1.35) | **0.002** |  | 1.46 (1.16 ~ 1.84) | **0.001** |  |
| No | 1.31 (1.21 ~ 1.43) | **<0.001** |  | 1.45 (1.23 ~ 1.72) | **<0.001** |  |
| Diabetes |  |  | 0.512 |  |  | 0.236 |
| Yes | 1.24 (1.04 ~ 1.49) | **0.018** |  | 1.19 (0.84 ~ 1.67) | 0.331 |  |
| No | 1.28 (1.19 ~ 1.38) | **<0.001** |  | 1.43 (1.21 ~ 1.70) | **<0.001** |  |
| Hypertension |  |  | 0.050 |  |  | 0.056 |
| Yes | 1.24 (1.14 ~ 1.35) | **<0.001** |  | 1.44 (1.20 ~ 1.72) | **<0.001** |  |
| No | 1.31 (1.19 ~ 1.44) | **<0.001** |  | 1.48 (1.16 ~ 1.89) | **0.002** |  |
| CKM |  |  | 0.870 |  |  | 0.233 |
| Stage 0 | 1.27 (0.74 ~ 2.17) | 0.389 |  | 0.17 (0.03 ~ 0.81) | **0.026** |  |
| Stage 1 | 1.21 (0.97 ~ 1.51) | 0.095 |  | 1.09 (0.68 ~ 1.74) | 0.732 |  |
| Stage 2 | 1.28 (1.19 ~ 1.38) | **<0.001** |  | 1.49 (1.27 ~ 1.76) | **<0.001** |  |
| Stage 3 | 1.23 (1.04 ~ 1.45) | **0.014** |  | 1.42 (1.00 ~ 2.01) | 0.051 |  |

Bold font indicates statistically significant differences (P < 0.05)

HR, hazard ratio; CI, confidence interval;

The model was adjusted for age, sex, race, marital status, education, uric acid, serum creatinine, BUN, HDL-C, LDL-C, TG, FBG, HbA1c, smoking, drinking, hypertension, diabetes

**Table S12** Subgroup analysis of the association between WHtR and all-cause and cardiovascular mortality in patients with CKM syndrome stage 0–3

| WHtR  per 0.1 higher | All-cause mortality | | | Cardiovascular mortality | | |
| --- | --- | --- | --- | --- | --- | --- |
|  | HR (95%CI) | *P* | *P* interaction | HR (95%CI) | *P* | *P* interaction |
| Age |  |  | **<0.001** |  |  | 0.022 |
| <60 years | 1.15 (1.02 ~ 1.30) | **0.021** |  | 1.55 (1.19 ~ 2.02) | **0.001** |  |
| ≥ 60 years | 1.02 (0.94 ~ 1.11) | 0.637 |  | 1.17 (0.97 ~ 1.43) | 0.106 |  |
| Gender |  |  | **0.011** |  |  | **0.010** |
| Male | 1.22 (1.09 ~ 1.37) | **<0.001** |  | 1.56 (1.27 ~ 1.91) | **<0.001** |  |
| Female | 1.00 (0.90 ~ 1.11) | 0.986 |  | 1.03 (0.82 ~ 1.30) | 0.794 |  |
| Smoking |  |  | 0.721 |  |  | 0.121 |
| Yes | 1.07 (0.94 ~ 1.22) | 0.286 |  | 1.78 (1.33 ~ 2.38) | **<0.001** |  |
| No | 1.10 (1.02 ~ 1.20) | 0.016 |  | 1.22 (1.00 ~ 1.47) | **0.047** |  |
| Drinking |  |  | 0.022 |  |  | 0.164 |
| Yes | 1.00 (0.88 ~ 1.13) | 0.952 |  | 1.12 (0.89 ~ 1.41) | 0.342 |  |
| No | 1.17 (1.06 ~ 1.28) | **<0.001** |  | 1.42 (1.16 ~ 1.73) | **<0.001** |  |
| Diabetes |  |  | 0.512 |  |  | 0.113 |
| Yes | 1.10 (0.90 ~ 1.34) | 0.340 |  | 0.96 (0.70 ~ 1.32) | 0.790 |  |
| No | 1.10 (1.02 ~ 1.19) | **0.016** |  | 1.34 (1.09 ~ 1.64) | **0.005** |  |
| Hypertension |  |  | 0.050 |  |  | 0.559 |
| Yes | 1.10 (1.00 ~ 1.22) | 0.053 |  | 1.37 (1.11 ~ 1.70) | **0.003** |  |
| No | 1.09 (0.99 ~ 1.21) | 0.084 |  | 1.24 (0.98 ~ 1.57) | 0.072 |  |
| CKM |  |  | 0.677 |  |  | 0.126 |
| Stage 0 | 1.20 (0.49 ~ 2.94) | 0.697 |  | 0.00 (0.01 ~ 0.01) | <0.001 |  |
| Stage 1 | 1.13 (0.88 ~ 1.45) | 0.327 |  | 1.22 (0.79 ~ 1.87) | 0.371 |  |
| Stage 2 | 1.10 (1.01 ~ 1.20) | **0.023** |  | 1.32 (1.09 ~ 1.60) | **0.004** |  |
| Stage 3 | 1.05 (0.90 ~ 1.24) | 0.526 |  | 1.29 (0.91 ~ 1.84) | 0.148 |  |

Bold font indicates statistically significant differences (P < 0.05)

HR, hazard ratio; CI, confidence interval;

The model was adjusted for age, sex, race, marital status, education, uric acid, serum creatinine, BUN, HDL-C, LDL-C, TG, FBG, HbA1c, smoking, drinking, hypertension, diabetes

**Table S13** Subgroup analysis of the association between BMI and all-cause and cardiovascular mortality in patients with CKM syndrome stage 0–3

| BMI  per 1 higher | All-cause mortality | | | Cardiovascular mortality | | |
| --- | --- | --- | --- | --- | --- | --- |
|  | HR (95%CI) | *P* | *P* interaction | HR (95%CI) | *P* | *P* interaction |
| Age |  |  | **<0.001** |  |  | 0.059 |
| <60 years | 1.01 (0.99 ~ 1.03) | 0.389 |  | 1.03 (0.99 ~ 1.07) | 0.104 |  |
| ≥ 60 years | 0.98 (0.97 ~ 0.99) | **0.002** |  | 1.00 (0.97 ~ 1.03) | 0.954 |  |
| Gender |  |  | **0.023** |  |  | **0.009** |
| Male | 1.01 (0.99 ~ 1.03) | 0.322 |  | 1.04 (1.01 ~ 1.08) | **0.012** |  |
| Female | 0.98 (0.97 ~ 1.00) | **0.016** |  | 0.98 (0.95 ~ 1.01) | 0.183 |  |
| Smoking |  |  | 0.176 |  |  | 0.111 |
| Yes | 0.99 (0.98 ~ 1.01) | 0.287 |  | 1.05 (1.01 ~ 1.09) | **0.021** |  |
| No | 1.00 (0.98 ~ 1.02) | 0.741 |  | 1.01 (0.98 ~ 1.04) | 0.655 |  |
| Drinking |  |  | **0.049** |  |  | 0.156 |
| Yes | 0.98 (0.96 ~ 1.00) | **0.033** |  | 0.99 (0.95 ~ 1.03) | 0.592 |  |
| No | 1.00 (0.99 ~ 1.02) | 0.734 |  | 1.02 (0.99 ~ 1.06) | 0.129 |  |
| Diabetes |  |  | 0.196 |  |  | 0.242 |
| Yes | 1.01 (0.98 ~ 1.03) | 0.722 |  | 0.98 (0.93 ~ 1.02) | 0.305 |  |
| No | 0.99 (0.98 ~ 1.00) | 0.129 |  | 1.02 (0.99 ~ 1.05) | 0.211 |  |
| Hypertension |  |  | 0.588 |  |  | 0.252 |
| Yes | 1.00 (0.98 ~ 1.01) | 0.753 |  | 1.02 (0.99 ~ 1.05) | 0.200 |  |
| No | 0.99 (0.97 ~ 1.00) | 0.153 |  | 1.00 (0.97 ~ 1.03) | 0.931 |  |
| CKM |  |  | 0.878 |  |  | 0.170 |
| Stage 0 | 1.04 (0.86 ~ 1.24) | 0.708 |  | 0.42 (0.34 ~ 0.54) | **<0.001** |  |
| Stage 1 | 1.01 (0.97 ~ 1.04) | 0.767 |  | 1.02 (0.97 ~ 1.07) | 0.505 |  |
| Stage 2 | 0.99 (0.98 ~ 1.01) | 0.331 |  | 1.01 (0.98 ~ 1.04) | 0.356 |  |
| Stage 3 | 0.99 (0.96 ~ 1.02) | 0.472 |  | 1.00 (0.95 ~ 1.06) | 0.904 |  |

Bold font indicates statistically significant differences (P < 0.05)

HR, hazard ratio; CI, confidence interval;

The model was adjusted for age, sex, race, marital status, education, uric acid, serum creatinine, BUN, HDL-C, LDL-C, TG, FBG, HbA1c, smoking, drinking, hypertension, diabetes

**Table S14**. Sensitivity analysis was performed using the Fine-Gray competing risk model, with non-cardiovascular death considered as a competing risk event.

| Variable | Model 1  SHR (95% CI) | *P* value | Model 2  SHR (95% CI) | *P* value | Model3  SHR (95% CI) | *P* value |
| --- | --- | --- | --- | --- | --- | --- |
| **BRI** |  |  |  |  |  |  |
| Per 1 higher | 1.14 (1.10 - 1.17) | **<0.001** | 1.08 (1.04 - 1.12) | **<0.001** | 1.05 (1.01 - 1.09) | **0.033** |
| Tertile 1 | 1.00 (Reference) |  | 1.00 (Reference) |  | 1.00 (Reference) |  |
| Tertile 2 | 1.35 (1.15 - 1.59) | **<0.001** | 0.91 (0.77 - 1.09) | 0.312 | 0.94 (0.79 - 1.12) | 0.475 |
| Tertile 3 | 1.76 (1.51 - 2.04) | **<0.001** | 0.90 (0.77 - 1.05) | 0.170 | 0.89 (0.76 - 1.05) | 0.177 |
| Tertile 4 | 2.51 (2.19 - 2.89) | **<0.001** | 1.28 (1.10 - 1.50) | **0.002** | 1.11 (0.92 - 1.34) | 0.261 |
| **ABSI** |  |  |  |  |  |  |
| Per 5 higher | 2.20 (2.07 - 2.34) | **<0.001** | 1.47 (135 - 1.60) | **<0.001** | 1.35 (1.23 - 1.47) | **<0.001** |
| Tertile 1 | 1.00 (Reference) |  | 1.00 (Reference) |  | 1.00 (Reference) |  |
| Tertile 2 | 1.49 (1.19 - 1.87) | **<0.001** | 1.21 (0.97 - 1.51) | 0.092 | 1.16 (0.93 - 1.44) | 0.190 |
| Tertile 3 | 2.54 (2.04 - 3.15) | **<0.001** | 1.55 (1.25 - 1.93) | **<0.001** | 1.42 (1.15 - 1.77) | **0.001** |
| Tertile 4 | 6.64 (5.60 - 7.87) | **<0.001** | 2.36 (1.98 - 2.82) | **<0.001** | 1.91 (1.61 - 2.28) | **<0.001** |
| **WWI** |  |  |  |  |  |  |
| Per 1 higher | 2.35 (2.14 - 2.57) | **<0.001** | 1.65(1.48- 1.84) | **<0.001** | 1.46 ( 1.29-1.65) | **<0.001** |
| Tertile 1 | 1.00 (Reference) |  | 1.00 (Reference) |  | 1.00 (Reference) |  |
| Tertile 2 | 1.81 (1.51 - 2.17) | **<0.001** | 1.27 (1.06 - 1.51) | **0.010** | 1.27 (1.06 - 1.51) | **0.009** |
| Tertile 3 | 2.93 (2.51 - 3.41) | **<0.001** | 1.50 (1.26 - 1.79) | **<0.001** | 1.46 (1.22 - 1.75) | **<0.001** |
| Tertile 4 | 6.28 (5.36 - 7.36) | **<0.001** | 2.25 (1.88 - 2.69) | **<0.001** | 1.93 (1.59 - 2.35) | **<0.001** |
| **WHtR** |  |  |  |  |  |  |
| Per 0.1 higher | 1.39 (1.28 - 1.50) | **<0.001** | 1.18 (1.07 - 1.30) | **<0.001** | 1.11 (1.02 - 1.22) | **0.037** |
| Tertile 1 | 1.00 (Reference) |  | 1.00 (Reference) |  | 1.00 (Reference) |  |
| Tertile 2 | 1.35 (1.15 - 1.59) | **<0.001** | 0.91 (0.77 - 1.09) | 0.312 | 0.94 (0.79 - 1.12) | 0.475 |
| Tertile 3 | 1.76 (1.51 - 2.04) | **<0.001** | 0.90 (0.77 - 1.05) | 0.170 | 0.89 (0.76 - 1.05) | 0.177 |
| Tertile 4 | 2.51 (2.19 - 2.89) | **<0.001** | 1.28 (1.10 - 1.50) | **0.002** | 1.11 (0.92 - 1.34) | 0.261 |
| **C-index** |  |  |  |  |  |  |
| Per 0.1 higher | 2.20 (2.03 - 2.38) | **<0.001** | 1.41 (1.28 - 1.56) | **<0.001** | 1.28 (1.14 - 1.42) | **<0.001** |
| Tertile 1 | 1.00 (Reference) |  | 1.00 (Reference) |  | 1.00 (Reference) |  |
| Tertile 2 | 1.60 (1.31 - 1.96) | **<0.001** | 1.18 (0.97 - 1.44) | 0.092 | 1.17 (0.95 - 1.43) | 0.134 |
| Tertile 3 | 2.69 (2.24 - 3.22) | **<0.001** | 1.42 (1.17 - 1.72) | **<0.001** | 1.37 (1.13 - 1.66) | **0.001** |
| Tertile 4 | 5.27 (4.55 - 6.11) | **<0.001** | 1.87 (1.58 - 2.22) | **<0.001** | 1.58 (1.33 - 1.88) | **<0.001** |
| **BMI** |  |  |  |  |  |  |
| Per 1 higher | 1.00 (0.99 - 1.02) | 0.974 | 1.00 (0.98 - 1.01) | 0.772 | 0.99 (0.97 - 1.00) | 0.087 |
| BMI＜18.5kg/m^2^ | 1.00 (Reference) |  | 1.00 (Reference) |  | 1.00 (Reference) |  |
| 18.5≤BMI＜25kg/m^2^ | 0.54 (0.40 - 0.74) | **<0.001** | 0.43 (0.32 - 0.59) | **<0.001** | 0.53 (0.38 - 0.74) | **<0.001** |
| 25≤BMI＜30kg/m^2^ | 0.59 (0.44 - 0.79) | **<0.001** | 0.36 (0.26 - 0.49) | **<0.001** | 0.44 (0.31 - 0.62) | **<0.001** |
| BMI≥30kg/m^2^ | 0.60 (0.44 - 0.82) | **0.002** | 0.41 (0.30 - 0.55) | **<0.001** | 0.44 (0.31 - 0.62) | **<0.001** |

Model 1: No adjustments

Model 2:Adjusted for age, sex, race

Model 3: Adjusted for age, sex, race,marital status, education, uric acid,serum creatinine, BUN, HDL-C, LDL-C, TG,FBG,HbA1c, smoking, drinking, hypertension, diabetes

Abbreviations: SHR, sub-hazard ratio; CI, confidence interval. Other abbreviations, see Table 1.

**Table S15.** Sensitivity analyses were performed after excluding participants who developed all-cause mortality within the first two years of follow-up.

| Variable | Model 1  HR (95% CI) | *P* value | Model 2  HR (95% CI) | *P* value | Model3  HR (95% CI) | *P* value |
| --- | --- | --- | --- | --- | --- | --- |
| **All-cause mortality** |  |  |  |  |  |  |
| **BRI** |  |  |  |  |  |  |
| Per 1 higher | 1.14 (1.12 - 1.17) | **<0.001** | 1.07 (1.04 - 1.10) | **<0.001** | 1.04 (1.01 - 1.08) | **0.011** |
| Tertile 1 | 1.00 (Reference) |  | 1.00 (Reference) |  | 1.00 (Reference) |  |
| Tertile 2 | 1.35 (1.15 - 1.59) | **<0.001** | 0.91 (0.77 - 1.09) | 0.312 | 0.94 (0.79 - 1.12) | 0.475 |
| Tertile 3 | 1.76 (1.51 - 2.04) | **<0.001** | 0.90 (0.77 - 1.05) | 0.170 | 0.89 (0.76 - 1.05) | 0.177 |
| Tertile 4 | 2.51 (2.19 - 2.89) | **<0.001** | 1.28 (1.10 - 1.50) | **0.002** | 1.11 (0.92 - 1.34) | 0.261 |
| **ABSI** |  |  |  |  |  |  |
| Per 5 higher | 2.11 (1.92 - 2.31) | **<0.001** | 1.42 (1.34 - 1.52) | **<0.001** | 1.30 (1.23 - 1.38) | **<0.001** |
| Tertile 1 | 1.00 (Reference) |  | 1.00 (Reference) |  | 1.00 (Reference) |  |
| Tertile 2 | 1.49 (1.19 - 1.87) | **<0.001** | 1.21 (0.97 - 1.51) | 0.092 | 1.16 (0.93 - 1.44) | 0.190 |
| Tertile 3 | 2.54 (2.04 - 3.15) | **<0.001** | 1.55 (1.25 - 1.93) | **<0.001** | 1.42 (1.15 - 1.77) | **0.001** |
| Tertile 4 | 6.64 (5.60 - 7.87) | **<0.001** | 2.36 (1.98 - 2.82) | **<0.001** | 1.91 (1.61 - 2.28) | **<0.001** |
| **WWI** |  |  |  |  |  |  |
| Per 1 higher | 2.33 (2.19 - 2.47) | **<0.001** | 1.52 (1.41 - 1.64) | **<0.001** | 1.38 (1.28 - 1.49) | **<0.001** |
| Tertile 1 | 1.00 (Reference) |  | 1.00 (Reference) |  | 1.00 (Reference) |  |
| Tertile 2 | 1.92 (1.38 - 2.68) | **<0.001** | 1.18 (0.84 - 1.65) | 0.350 | 1.21 (0.86 - 1.70) | 0.276 |
| Tertile 3 | 2.90 (2.12- 3.98) | **<0.001** | 1.24 (0.89 - 1.72) | 0.197 | 1.19 (0.85 - 1.66) | 0.315 |
| Tertile 4 | 7.34 (5.50 - 9.80) | **<0.001** | 2.40 (1.76 - 3.28) | **<0.001** | 2.08 (1.50 - 2.89) | **<0.001** |
| **WHtR** |  |  |  |  |  |  |
| Per 0.1 higher | 1.40 (1.33 - 1.47) | **<0.001** | 1.16 (1.09 - 1.24) | **<0.001** | 1.09 (1.01 - 1.18) | **0.032** |
| Tertile 1 | 1.00 (Reference) |  | 1.00 (Reference) |  | 1.00 (Reference) |  |
| Tertile 2 | 1.35 (1.15 - 1.59) | **<0.001** | 0.91 (0.77 - 1.09) | 0.312 | 0.94 (0.79 - 1.12) | 0.475 |
| Tertile 3 | 1.76 (1.51 - 2.04) | **<0.001** | 0.90 (0.77 - 1.05) | 0.170 | 0.89 (0.76 - 1.05) | 0.177 |
| Tertile 4 | 2.51 (2.19 - 2.89) | **<0.001** | 1.28 (1.10 - 1.50) | **0.002** | 1.11 (0.92 - 1.34) | 0.261 |
| **C-index** |  |  |  |  |  |  |
| Per 0.1 higher | 2.05 (1.92 - 2.19) | **<0.001** | 1.37 (1.28 - 1.47) | **<0.001** | 1.26 (1.18 - 1.35) | **<0.001** |
| Tertile 1 | 1.00 (Reference) |  | 1.00 (Reference) |  | 1.00 (Reference) |  |
| Tertile 2 | 1.77 (1.26 - 2.50) | **0.001** | 1.15 (0.81 - 1.63) | 0.436 | 1.15 (0.81 - 1.63) | 0.436 |
| Tertile 3 | 3.29 (2.40 - 4.50) | **<0.001** | 1.41 (1.02- 1.95) | **0.038** | 1.37 (0.98 - 1.91) | 0.066 |
| Tertile 4 | 7.28 (5.43 - 9.77) | **<0.001** | 2.06 (1.50 - 2.81) | **<0.001** | 1.75 (1.26 - 2.43) | **<0.001** |
| **BMI** |  |  |  |  |  |  |
| Per 1 higher | 1.01 (1.00 - 1.02) | 0.201 | 1.00 (0.99 - 1.01) | 0.876 | 0.99 (0.98 - 1.01) | 0.320 |
| BMI＜18.5kg/m^2^ | 1.00 (Reference) |  | 1.00 (Reference) |  | 1.00 (Reference) |  |
| 18.5≤BMI＜25kg/m^2^ | 0.79 (0.39 - 1.61) | 0.525 | 0.51 (0.25 - 1.03) | 0.061 | 0.65 (0.32 - 1.34) | 0.244 |
| 25≤BMI＜30kg/m^2^ | 0.94 (0.46 - 1.90) | 0.858 | 0.44 (0.22 - 0.89) | 0.023 | 0.55 (0.27 - 1.12) | 0.100 |
| BMI≥30kg/m^2^ | 0.83 (0.41 - 1.67) | 0.594 | 0.45 (0.22 - 0.92) | 0.028 | 0.49 (0.24 - 1.02) | 0.058 |

Model 1: No adjustments

Model 2:Adjusted for age, sex, race

Model 3: Adjusted for age, sex, race,marital status, education, uric acid,serum creatinine, BUN, HDL-C, LDL-C, TG,FBG,HbA1c, smoking, drinking, hypertension, diabetes

Abbreviations:HR, hazard ratio; CI, confidence interval. Other abbreviations, see Table 1.

**Table S16.** Sensitivity analyses were performed after excluding participants who developed Cardiovascular mortality within the first two years of follow-up.

| Variable | Model 1  HR (95% CI) | *P* value | Model 2  HR (95% CI) | *P* value | Model3  HR (95% CI) | *P* value |
| --- | --- | --- | --- | --- | --- | --- |
| **Cardiovascular mortality** |  | | | | | |
| **BRI** |  |  |  |  |  |  |
| Per 1 higher | 1.21 (1.17 - 1.26) | **<0.001** | 1.17 (1.10 - 1.24) | **<0.001** | 1.13 (1.05 - 1.21) | **<0.001** |
| Tertile 1 | 1.00 (Reference) |  | 1.00 (Reference) |  | 1.00 (Reference) |  |
| Tertile 2 | 1.42 (1.01 - 2.00) | **0.046** | 0.90 (0.63 - 1.27) | 0.546 | 0.93 (0.65 - 1.32) | 0.670 |
| Tertile 3 | 2.08 (1.50 - 2.89) | **<0.001** | 0.95 (0.67 - 1.36) | 0.784 | 0.93 (0.63 - 1.35) | 0.689 |
| Tertile 4 | 3.72 (2.65 - 5.23) | **<0.001** | 1.74 (1.20 - 2.50) | **0.003** | 1.44 (0.93 - 2.23) | 0.103 |
| **ABSI** |  |  |  |  |  |  |
| Per 5 higher | 2.27 (2.01 - 2.57) | **<0.00** | 1.48 (1.31 - 1.68) | **<0.00** | 1.36 (1.20 - 1.55) | **<0.00** |
| Tertile 1 | 1.00 (Reference) |  | 1.00 (Reference) |  | 1.00 (Reference) |  |
| Tertile 2 | 1.83 (1.20 - 2.79) | **0.005** | 1.41 (0.91 - 2.20) | 0.125 | 1.37 (0.90 - 2.09) | 0.147 |
| Tertile 3 | 2.79 (1.74 - 4.47) | **<0.001** | 1.54 (0.91 - 2.60) | 0.106 | 1.42 (0.85 - 2.38) | 0.186 |
| Tertile 4 | 9.42 (6.42 - 13.82) | **<0.001** | 2.78 (1.72 - 4.49) | **<0.001** | 2.28 (1.43 - 3.64) | **<0.001** |
| **WWI** |  |  |  |  |  |  |
| Per 1 higher | 2.80 (2.48 - 3.16) | **<0.001** | 1.85 (1.56 - 2.19) | **<0.001** | 1.66 (1.38 - 2.01) | **<0.001** |
| Tertile 1 | 1.00 (Reference) |  | 1.00 (Reference) |  | 1.00 (Reference) |  |
| Tertile 2 | 2.18 (1.45 - 3.29) | **<0.001** | 1.47 (0.94 - 2.28) | 0.088 | 1.48 (0.94 - 2.35) | 0.093 |
| Tertile 3 | 3.88 (2.60 - 5.78) | **<0.001** | 1.86 (1.16 - 2.96) | **0.009** | 1.87 (1.14 - 3.05) | **0.012** |
| Tertile 4 | 10.59 (7.01 - 16.01) | **<0.001** | 3.48 (2.06 - 5.86) | **<0.001** | 3.00 (1.70 - 5.32) | **<0.001** |
| **WHtR** |  |  |  |  |  |  |
| Per 0.1 higher | 1.65 (1.48 - 1.83) | **<0.001** | 1.44 (1.23 - 1.68) | **<0.001** | 1.32 (1.09 - 1.59) | **0.004** |
| Tertile 1 | 1.00 (Reference) |  | 1.00 (Reference) |  | 1.00 (Reference) |  |
| Tertile 2 | 1.42 (1.01 - 2.00) | **0.046** | 0.90 (0.63 - 1.27) | 0.546 | 0.93 (0.65 - 1.32) | 0.670 |
| Tertile 3 | 2.08 (1.50 - 2.89) | **<0.001** | 0.95 (0.67 - 1.36) | 0.784 | 0.93 (0.63 - 1.35) | 0.689 |
| Tertile 4 | 3.72 (2.65 - 5.23) | **<0.001** | 1.74 (1.20 - 2.50) | **0.003** | 1.44 (0.93 - 2.23) | 0.103 |
| **C-index** |  |  |  |  |  |  |
| Per 0.1 higher | 2.42 (2.12 - 2.76) | **<0.001** | 1.58 (1.36 - 1.84) | **<0.001** | 1.43 (1.22 - 1.69) | **<0.001** |
| Tertile 1 | 1.00 (Reference) |  | 1.00 (Reference) |  | 1.00 (Reference) |  |
| Tertile 2 | 1.98 (1.40 - 2.80) | **<0.001** | 1.40 (0.97 - 2.04) | 0.076 | 1.38 (0.95 - 2.01) | 0.094 |
| Tertile 3 | 3.38 (2.25 - 5.08) | **<0.001** | 1.61 (1.01 - 2.57) | **0.044** | 1.58 (0.99 - 2.52) | 0.055 |
| Tertile 4 | 9.12 (6.19 - 13.44) | **<0.001** | 2.80 (1.78 - 4.43) | **<0.001** | 2.31 (1.44 - 3.70) | **<0.001** |
| **BMI** |  |  |  |  |  |  |
| Per 1 higher | 1.03 (1.01 - 1.05) | **0.002** | 1.03 (1.01 - 1.05) | **0.013** | 1.02 (0.99 - 1.04) | 0.202 |
| BMI＜18.5kg/m^2^ | 1.00 (Reference) |  | 1.00 (Reference) |  | 1.00 (Reference) |  |
| 18.5≤BMI＜25kg/m^2^ | 0.52 (0.25 - 1.11) | 0.090 | 0.41 (0.20 - 0.84) | **0.015** | 0.49 (0.23 - 1.01) | 0.054 |
| 25≤BMI＜30kg/m^2^ | 0.70 (0.36 - 1.37) | 0.301 | 0.40 (0.21 - 0.76) | **0.005** | 0.46 (0.24 - 0.90) | **0.023** |
| BMI≥30kg/m^2^ | 0.82 (0.41 - 1.64) | 0.582 | 0.53 (0.28 - 1.01) | 0.055 | 0.51 (0.26 - 1.01) | 0.054 |

Model 1: No adjustments

Model 2:Adjusted for age, sex, race

Model 3: Adjusted for age, sex, race,marital status, education, uric acid,serum creatinine, BUN, HDL-C, LDL-C, TG,FBG,HbA1c, smoking, drinking, hypertension, diabetes

Abbreviations:HR, hazard ratio; CI, confidence interval. Other abbreviations, see Table 1.

**Table S17**. Predictive performance of models for all-cause mortality and cardiovascular mortality.

|  | IDI | P-value | Continuous NRI | P-value |
| --- | --- | --- | --- | --- |
|  | Estimate (95%CI) |  | Estimate (95%CI) |  |
| **All-cause mortality** |  |  |  |  |
| Model 3 | Reference |  | Reference |  |
| Model 3+WWI | 0.0356(0.0193-0.0569) | **<0.001** | 0.1831(0.1289-0.1992) | **<0.001** |
| Model 3+ABSI | 0.0572(0.0365-0.0839) | **<0.001** | 0.2191(0.1644-0.2877) | **<0.001** |
| Model 3+C-index | 0.0245(0.0118-0.0432) | **<0.001** | 0.1805(0.1173-0.2398) | **<0.001** |
| Model 3+WHtR | -0.0001(-0.0003-0.0012) | 0.613 | -0.0418(-0.0593-0.0689) | 0.800 |
| Model 3+BRI | 0.0003(-0.0005-0.0042) | **<0.001** | -0.0287(-0.0673-0.0099) | 0.145 |
| Model 3+BMI | 0.0104(0.0017- 0.0259) | **0.006** | 0.0765(0.0111-0.1370) | **0.022** |
| **Cardiovascular mortality** |  |  |  |  |
| Model 3 | Reference |  | Reference |  |
| Model 3+WWI | 0.0507(0.0303-0.0726) | **<0.001** | 0.2407(0.1452-0.3063) | **<0.001** |
| Model 3+ABSI | 0.0505(0.0310-0.0785) | **<0.001** | 0.2355(0.1659-0.3259) | **<0.001** |
| Model 3+C-index | 0.0286(0.0076, 0.0557) | **<0.001** | 0.1990(0.0764, 0.3067) | **<0.001** |
| Model 3+WHtR | 0.0045(-0.0003-0.0184) | 0.200 | 0.1990(-0.0525-0.1461) | 0.400 |
| Model 3+BRI | 0.0073(0.0001-0.0241) | **<0.001** | 0.0648(-0.0652-0.1695) | 0.600 |
| Model 3+BMI | 0.0012(-0.0029-0.0227) | 0.500 | 0.0491(-0.1143-0.0934) | 0.400 |

Abbreviations: IDI, integrated discrimination improvement; NRI, net reclassification index;

Other abbreviations, see Table 1.

# 3 Supplemental figures


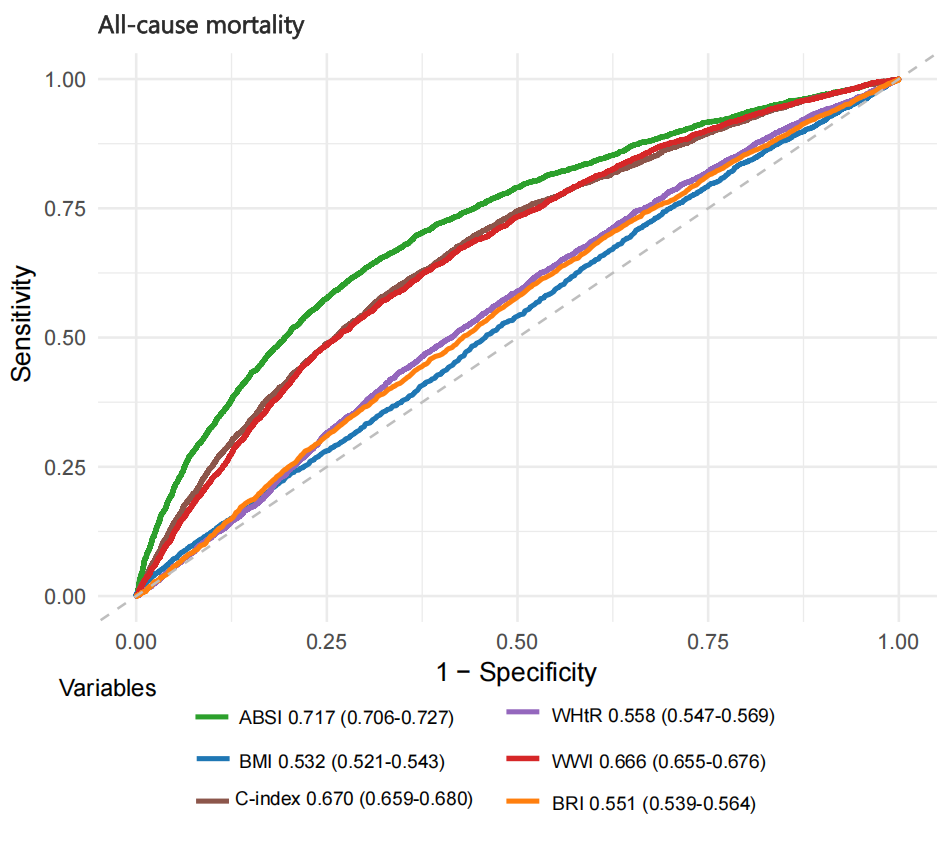


**Figure S1** ROC curve analysis of the predictive value for All-cause mortality outcome with the BMI, ABSI, WHtR, WWI, C-index, and BRI. For other abbreviations, see Table 1.


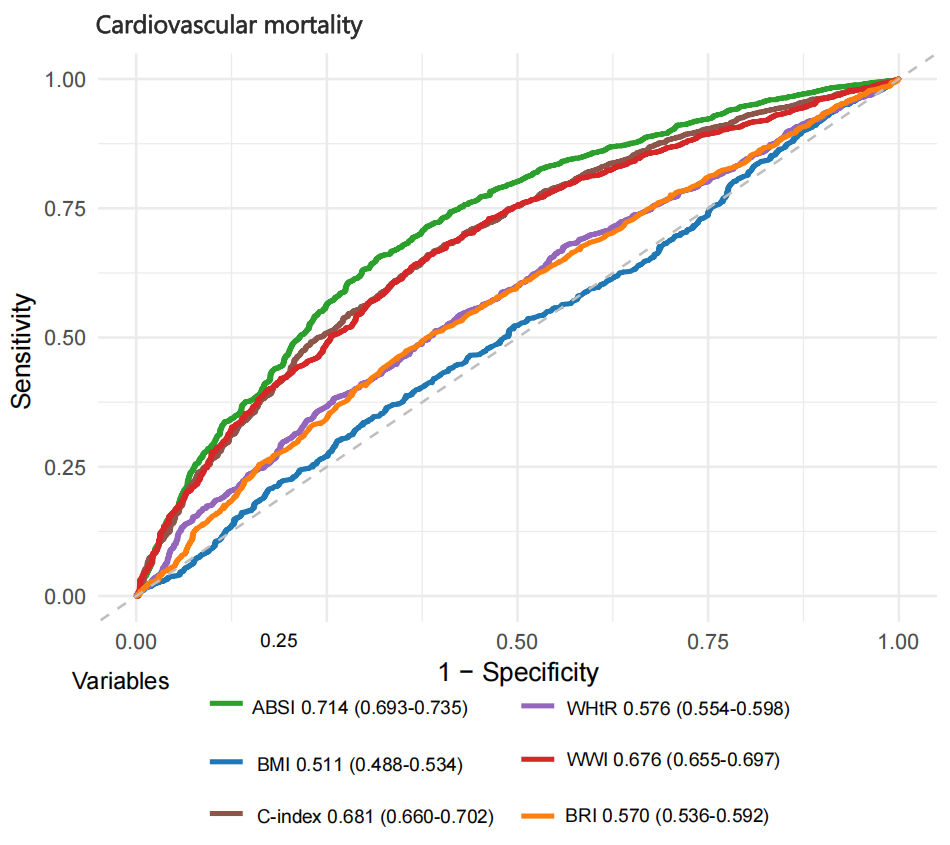


**Figure S2** ROC curve analysis of the predictive value for Cardiovascular mortality outcome with the BMI, ABSI, WHtR, WWI, C-index, and BRI. For other abbreviations, see Table 1.

**References**

[1] Ndumele CE, Neeland IJ, Tuttle KR, et al. A Synopsis of the Evidence for the Science and Clinical Management of Cardiovascular-Kidney-Metabolic (CKM) Syndrome: A Scientific Statement From the American Heart Association. Circulation. 2023. 148(20): 1636-1664.

[2] Kidney Disease: Improving Global Outcomes (KDIGO) CKD Work Group, . KDIGO 2024 Clinical Practice Guideline for the Evaluation and Management of Chronic Kidney Disease. Kidney Int. 2024. 105(4S): S117-S314.

[3] Inker LA, Eneanya ND, Coresh J, et al. New Creatinine- and Cystatin C-Based Equations to Estimate GFR without Race. N Engl J Med. 2021. 385(19): 1737-1749.

[4] Sr DRB, Vasan RS, Pencina MJ, et al. General cardiovascular risk profile for use in primary care: the Framingham Heart Study. Circulation. 2008. 117(6): 743-53.
